# Relative-Height Image Generation from Long-Range Airborne Streak-Tube Imaging LiDAR for Wide-Area Building- Structure Mapping

**DOI:** 10.3390/jimaging12080355

**Published:** 2026-08-04

**Authors:** Chaowei Dong, Zhaodong Chen, Rongwei Fan, Zhiwei Dong, Deying Chen, Pengfei Hao, Lansong Cao

**Affiliations:** 1 National Key Laboratory of Laser Spatial Information, Harbin Institute of Technology, Harbin 150001, China; 2 Space Representative Office, Changchun 130000, China

**Keywords:** airborne streak-tube imaging LiDAR, ASTIL, streak-tube detector, spatial–temporal echo imaging, long-range remote sensing, wide-area remote sensing, relative-height imaging, building-structure mapping

## Abstract

Wide-area building-structure mapping from long-range airborne LiDAR requires image products that can represent building footprints, roof-height variations, and structural discontinuities with low computational latency. Airborne streak-tube imaging LiDAR (ASTIL) records a spatial–temporal echo image for each laser pulse, where the detector row corresponds to the fan-beam spatial angle, the detector column encodes echo arrival time, and the frame sequence represents the scanning process. This row–column–frame topology makes it possible to generate image-domain structural products directly from raw streak-tube echo sequences. In this paper, a relative-height image generation method is proposed for long-range ASTIL. The method constructs slant-range matrices from raw echo images, suppresses row-wise ground-related range trends, maps the residuals into relative-height values, and generates scan-geometry-calibrated swath-level relative-height images using lightweight calibration rather than rigorous point-wise POS/IMU trajectory reconstruction. Airborne experiments at 2 km, 3 km, and 6 km flight heights show that the proposed workflow can generate relative-height images with spatial sampling intervals of 0.30 m, 0.45 m, and 0.90 m, respectively, within a 0.5 s acquisition window. The generated cropped image products occupy less than 0.3% of the raw streak-image sequence volume, reflecting a compact image-domain representation for rapid preliminary mapping rather than lossless data compression. Building-scale comparisons with UAV LiDAR reference data indicate that the generated images preserve the main building footprints, boundary orientations, and roof-height discontinuities. For nine flat-roof targets, the mean absolute roof-to-ground height errors range from 0.24 m to 0.30 m across the three flight heights. These results suggest that ASTIL relative-height imaging can provide an efficient image-domain representation for wide-area building-structure mapping under long-range airborne observation conditions.

## 1. Introduction

Wide-area building-structure mapping is an important task in airborne remote sensing, urban infrastructure monitoring, and regional disaster assessment. For large built-up areas, information on building footprints, roof structures, height discontinuities, and spatial layouts is useful for rapid scene interpretation and subsequent detailed inspection. In such applications, a dense and fully georeferenced point cloud is not always required at the first processing stage. Instead, a compact image-domain product that rapidly represents building-scale spatial and height information can provide an efficient intermediate representation for preliminary wide-area analysis.

Airborne LiDAR has been widely used to acquire three-dimensional structural information over urban and natural environments [1,2]. Conventional airborne LiDAR workflows generally reconstruct georeferenced point clouds before generating digital elevation models, range images, or other raster products [3,4]. Recent studies on airborne LiDAR point-cloud classification, building extraction, roof-contour extraction, and building reconstruction further demonstrate the importance of point-cloud-based urban mapping [5,6,7,8,9]. At the same time, modern point-cloud processing increasingly uses graph neural networks and Transformer-based architectures to improve structural representation and semantic interpretation [5,10,11]. These methods are powerful, but they usually require large point sets, neighborhood construction, feature aggregation, pose-aware registration, and substantial memory or computation for large-scale scenes. Therefore, for rapid preliminary mapping from long-range airborne observations, it remains useful to investigate image-domain representations that can reduce the dependence on dense point-cloud-first processing.

LiDAR image representations have been studied to improve processing efficiency and compatibility with image-processing algorithms. Existing methods commonly rely on point-cloud projection, rasterization, interpolation, range-view representation, or hardware-assisted range-image generation [12,13,14,15]. Recent range-view and cylindrical/spherical projection studies further show that native or projection-based LiDAR images can support efficient segmentation, rendering, and in-stream processing, although projection and rasterization may introduce quantization loss or discard part of the original 3-D measurements [16,17,18]. These studies demonstrate the value of image-domain LiDAR processing, but most of them still treat the point cloud as the primary intermediate product and then convert it into an image representation.

Airborne streak-tube imaging LiDAR (ASTIL) has a different data structure from conventional scanning LiDAR. For each emitted laser pulse, the streak-tube detector records both the spatial distribution along the fan-shaped laser beam and the echo arrival time within a single exposure [19]. As a result, each raw streak image has an intrinsic row–column structure: the row direction corresponds to the fan-beam spatial angle, while the column direction corresponds to echo time and range. Combined with the frame sequence generated by mechanical scanning, ASTIL data naturally form a row–column–frame topology. Previous studies have demonstrated ASTIL single-echo target extraction, ground target extraction, high-accuracy reconstruction, long-range system validation, and streak-image time–space calibration [20,21,22,23,24]. However, direct relative-height image generation from this intrinsic ASTIL data topology has not been sufficiently investigated.

The goal of this study is to determine whether the native row–column–frame topology of long-range ASTIL raw streak images can be used to generate compact relative-height image products for wide-area building-structure mapping without dense point-cloud-first reconstruction. To achieve this goal, four research tasks are addressed: (1) to formulate the ASTIL raw echo sequence as a matrix-domain spatial–temporal measurement cube; (2) to construct centroid, slant-range, and relative-height matrices directly from raw streak images; (3) to develop a lightweight scan-geometry calibration strategy for generating local swath-level relative-height images; and (4) to evaluate image-level coverage, data-volume compactness, building-boundary preservation, and relative roof-height accuracy using multi-altitude airborne experiments.

The main contributions of this study are summarized as follows:

1. The intrinsic row–column–frame topology of ASTIL raw streak images is analyzed for direct slant-range and relative-height image representation.
2. A relative-height image generation workflow is developed to transform raw echo image sequences into slant-range matrices, sensor-domain relative-height matrices, and calibrated swath-level relative-height images.
3. A lightweight scan-geometry calibration strategy is introduced by combining anisotropic scaling, projective correction, and flight-motion compensation under short-term stable flight conditions.
4. Multi-altitude airborne experiments at 2 km, 3 km, and 6 km are conducted to evaluate image-level acquisition efficiency, output data compactness, building-boundary preservation, and relative roof-height accuracy.

The proposed workflow is not intended to replace rigorous POS/IMU-based point-cloud reconstruction for high-precision surveying. Instead, it provides a compact image-domain representation for rapid wide-area building-structure mapping under long-range airborne observation conditions.

The remainder of this paper is organized as follows: Section 2 presents the materials and methods, including the ASTIL spatial–temporal imaging mechanism, scanning topology, relative-height image generation workflow, system configuration, and experimental data. Section 3 reports the image-level and building-scale results. Section 4 discusses the main findings, uncertainty sources, and applicability of the proposed image-domain representation based on the experimental results. Section 5 concludes the paper.

## 2. Materials and Methods

### 2.1. ASTIL Spatial–Temporal Imaging and Scanning Topology

#### 2.1.1. Spatial–Temporal Echo Imaging of the Streak-Tube Detector

**Figure 1:**
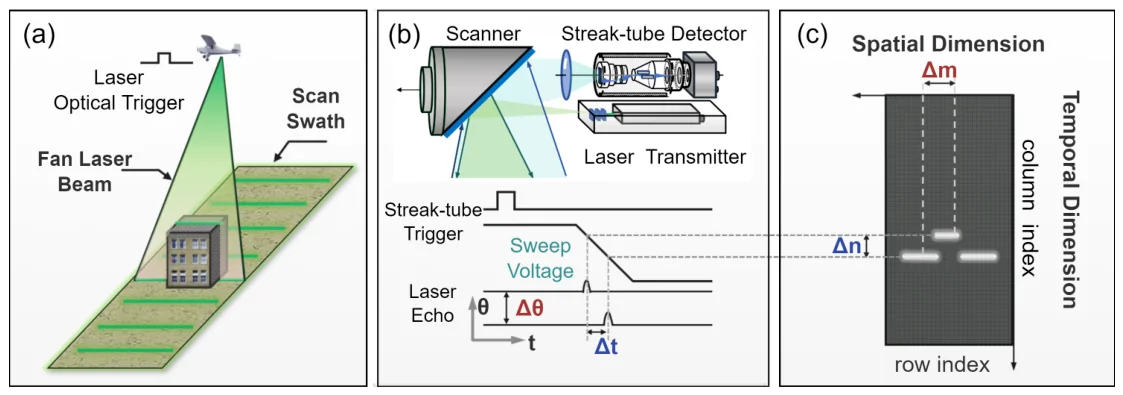
Spatial–temporal echo imaging principle of a single ASTIL streak image. (**a**) Laser emission by the airborne ASTIL system. (**b**) Echo reception and streak-tube imaging of the returned signal. (**c**) Acquired raw streak image, in which the detector row records the spatial distribution along the fan-shaped laser beam and the detector column records the echo arrival time. The echo column coordinate is converted into echo time, while the detector row coordinate is converted into spatial angle through linear calibration relationships.

Airborne streak-tube imaging LiDAR (ASTIL) records a one-dimensional spatial–temporal echo profile from a single laser pulse. The fan-shaped laser beam illuminates a ground profile, and the returned photons are imaged by the streak-tube detector. In a raw streak image, the detector row represents the spatial position along the fan beam, whereas the detector column represents the echo arrival time. Thus, a single streak image contains both the spatial distribution of the illuminated profile and the temporal waveform required for range measurement [19,24]. Figure 1 illustrates this spatial–temporal echo-imaging mechanism and the corresponding row-to-angle and column-to-time calibration relationships. The novelty considered here is not the time-of-flight ranging principle itself but, rather, the use of this native spatial–temporal encoding as the first level of a matrix-domain data structure for subsequent relative-height image formation.

The range measurement follows the time-of-flight principle:

(1) $$d=\frac{c\;t_{\mathrm{range}}}{2n_{\mathrm{air}}},$$

where *d* is the slant range, *c* is the speed of light in vacuum, $n_{\mathrm{air}}$ is the refractive index of air, and $t_{\mathrm{range}}$ is the round-trip propagation time. In ASTIL, the complete ranging time is formed by the TDC trigger timing, the echo time encoded by the streak-image column coordinate, and the calibrated system delay:

(2) $$t_{\mathrm{range}}=\Delta t_{\mathrm{trigger}}+t_{\mathrm{echo}}+\tau_{\mathrm{sys}},$$

where $\Delta t_{\mathrm{trigger}}$ is the TDC-recorded trigger delay, $t_{\mathrm{echo}}$ is the echo time within the streak image, and $\tau_{\mathrm{sys}}$ is the calibrated system delay.

For a single streak image, let *m* and *n* denote the detector row and column coordinates, respectively. If the extracted echo column coordinate is $n_{\mathrm{echo}}$, the column-to-time and row-to-angle relationships can be approximated as

(3) $$t_{\mathrm{echo}}=t_{0}+\alpha_{t}n_{\mathrm{echo}},$$

(4) $$\theta =\theta_{0}+\alpha_{\theta }m,$$

where $t_{0}$ and $\theta_{0}$ are calibration offsets, and $\alpha_{t}$ and $\alpha_{\theta }$ are the temporal and angular sampling coefficients. This linear expression is used as a first-order description of the detector sampling relationship; in the experiments, two-dimensional temporal and spatial-angle calibration matrices are used to account for residual time–space coupling in the streak-tube detector. An echo point extracted from a streak image can therefore be interpreted as a calibrated spatial-range sample. This single-frame encoding provides the basis for subsequent slant-range matrix construction: the column direction provides fine echo-time sampling, the row direction provides spatial-angle sampling, and the TDC trigger timing provides the coarse timing reference for long-range measurement.

#### 2.1.2. Airborne Scanning Geometry and Swath Pattern

**Figure 2:**
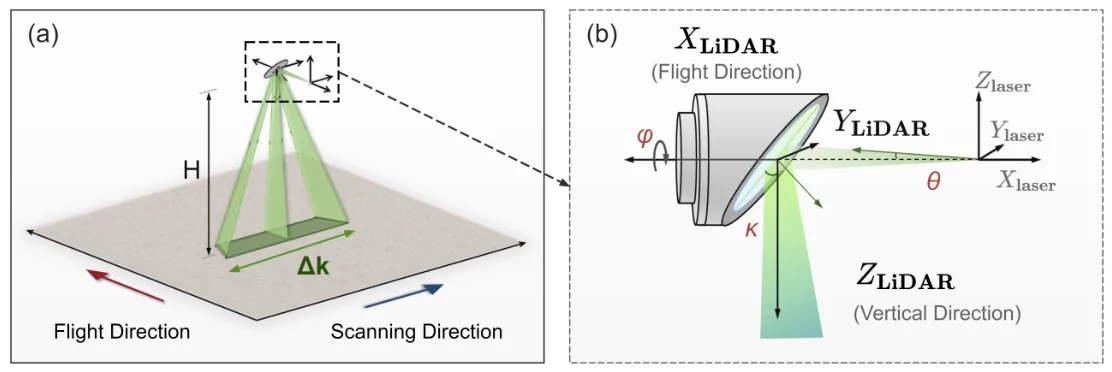
ASTIL scanning topology. (**a**) Schematic of the scanning emission process, in which the fan-beam laser is projected onto the ground while the scan mirror angle varies linearly. (**b**) Structural diagram of the scanning unit, showing the relationship among the detector row, detector column, fan-beam spatial angle θ, and scan angle ϕ(k). The detector row corresponds to the fan-beam spatial angle θ, the detector column provides echo-time information for range measurement, and the frame index corresponds to the linearly varying scan angle ϕ(k). The combination of single-frame spatial–temporal streak imaging and frame-wise mirror scanning forms a structured ground swath.

During airborne acquisition, the scanning mirror and the forward motion of the platform organize consecutive one-dimensional ASTIL echo profiles into a two-dimensional ground swath. A rigorous georeferencing solution requires platform position, attitude, installation parameters, and boresight calibration [25,26,27,28,29]. Here, an idealized scanning model is introduced to describe the dominant geometric relationship among the row-wise fan-beam angle, the frame-wise scan angle, and the resulting ground sampling pattern in the local LiDAR coordinate system. Figure 2 summarizes the ASTIL scanning topology formed by the fan-beam spatial angle, the echo-time column, and the frame-wise scan angle.

The frame index is converted into the mirror scan angle using a linear scanning relation:

(5) $$\phi \left(k\right)=\phi_{0}+\alpha_{\phi }\left(k-1\right),\;k=1,\ldots ,K,$$

where $\phi_{0}$ is the initial scan angle and $\alpha_{\phi }$ is the frame-to-scan-angle coefficient determined by the scanner’s angular sampling.

The incident laser direction before mirror reflection is represented as

(6) $$l_{\mathrm{in}}\left(\theta \right)=\frac{1}{\sqrt{1+\tan^{2}\left(\theta \right)}}\left[\begin{array}{c} -1\\ 0\\ \tan(\theta ) \end{array}\right].$$

The scanning mirror is modeled as an ideal plane mirror. Its unit normal vector is determined by the fixed mounting angle κ and the frame-dependent scan angle ϕ(k):

(7) $$n_{m}\left(k\right)=R_{x}\left(\phi (k)\right)v_{m}\left(\kappa \right),$$

where $R_{x}\left(\cdot \right)$ is the rotation matrix around the scanner’s *x*-axis and $v_{m}\left(\kappa \right)$ is the mirror normal at the nominal mounting angle. According to the plane-mirror reflection law, the outgoing laser direction is

(8) $$l_{\mathrm{out}}\left(\theta ,k\right)=l_{\mathrm{in}}\left(\theta \right)-2\left[l_{\mathrm{in}}^{T}\left(\theta \right)n_{m}\left(k\right)\right]n_{m}\left(k\right).$$

Thus, the instantaneous sampling direction is jointly determined by the single-frame fan-beam angle θ and the frame-wise scan angle ϕ(k).

Under the ideal assumption of a flat reference ground and a fixed flight height *H*, the intersection between the outgoing laser direction and the ground plane defines the instantaneous sampling position in the local LiDAR coordinate system. For the nominal mirror mounting angle $\kappa =45^{\circ }$, the sampling coordinates can be expressed as

(9) $$x_{\mathrm{LiDAR}}\left(\theta ,k\right)=\frac{\cos\left(\phi (k)\right)\tan\left(\theta \right)}{\cos\left(\phi (k)\right)+\sin^{2}\left(\phi (k)\right)\tan\left(\theta \right)}H,$$

(10) $$y_{\mathrm{LiDAR}}\left(\theta ,k\right)=\frac{-\sin\left(\phi (k)\right)-\sin\left(\phi (k)\right)\cos\left(\phi (k)\right)\tan\left(\theta \right)}{\cos\left(\phi (k)\right)+\sin^{2}\left(\phi (k)\right)\tan\left(\theta \right)}H.$$

Here, $x_{\mathrm{LiDAR}}$ and $y_{\mathrm{LiDAR}}$ denote the instantaneous ground sampling coordinates in the local LiDAR coordinate system. In this coordinate system, $x_{\mathrm{LiDAR}}$ is associated with the flight direction, whereas $y_{\mathrm{LiDAR}}$ is associated with the cross-track scanning direction. Equations (9) and (10) describe the dominant sampling geometry determined by the row-wise fan-beam angle θ and the frame-wise scan angle ϕ(k).

Within a single streak image, θ spans the spatial angular range of the fan-beam echo profile. Across consecutive frames, ϕ(k) samples the scanning direction according to the linear scanning coefficient $\alpha_{\phi }$. At kilometer-level flight heights, this row-wise fan-beam sampling and frame-wise mirror scanning form a wide ground swath in the local LiDAR coordinate system. Therefore, the continuous streak-image sequence contains the target echo intensity, echo-time information, and range-related measurements required for subsequent slant-range matrix construction and swath-level relative-height imaging.

### 2.2. Relative-Height Image Generation Method

**Figure 3:**
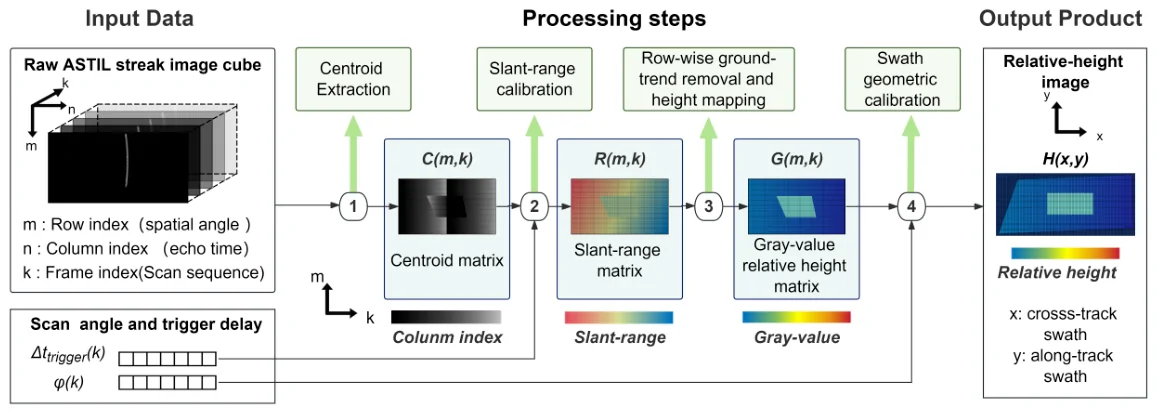
Topology-driven ASTIL relative-height image generation workflow. The raw ASTIL image cube and the scan/trigger-delay sequences are transformed into four core matrix products: the centroid matrix C, the slant-range matrix R, the gray-value height matrix G, and the final relative-height image H. Blue boxes denote data products, while green boxes denote processing operations.

Figure 3 illustrates the proposed topology-driven relative-height image generation workflow for airborne streak-tube imaging LiDAR (ASTIL). Unlike conventional LiDAR processing chains that convert echo measurements directly into three-dimensional point clouds, ASTIL records raw echoes as a row–column–frame image sequence with an intrinsic sensor topology [30]. In this topology, detector rows encode the spatial-angle dimension, detector columns encode echo time and range information, and consecutive frames describe the scanning process. Therefore, the raw ASTIL streak-image sequence is not only an intensity image sequence but also a sensor-topology-encoded measurement cube that jointly contains echo intensity, range timing, and scanning-geometry information.

From a software-implementation perspective, the proposed workflow stores and transforms the ASTIL measurements mainly as regular image matrices rather than as irregular point-cloud arrays. The raw input is an 8-bit image sequence ${\left\{I_{k}\right\}}_{k=1}^{K}$, the centroid matrix C stores sub-pixel echo-column coordinates, and the slant-range matrix R stores calibrated physical ranges. After row-wise ground-trend removal, the relative-height residuals are mapped into the gray-value height matrix G and then resampled into the final relative-height image H. The dense georeferenced point cloud is not an intermediate requirement of the proposed image-formation chain; it is generated only as a comparison product in the experiments. This matrix-domain data flow reduces the need for storing, transferring, and repeatedly interpolating large irregular point-cloud arrays during the preliminary image generation stage.

The raw ASTIL measurements are recorded as an 8-bit frame sequence ${\left\{I_{k}\right\}}_{k=1}^{K}$, where each raw streak image $I_{k}^{\mathrm{raw}}$ typically has a size of 512×1024 pixels and stores the echo intensity using 256 gray levels. During acquisition, the imaging gain is adjusted according to the operating range to ensure complete echo recording. Due to the effective field of view of the detector, only the valid echo rows are retained for subsequent processing. The effective streak image is denoted as $I_{k}\in {\left\{0,1,\ldots ,255\right\}}^{M\times N}$, where *M* is the number of effective echo channels and *N* is the number of temporal sampling columns. In the experiments, the effective image region is approximately 400×1024, i.e., M≈400 and N=1024.

Based on the row–column–frame topology of ASTIL, the proposed workflow generates four matrix-domain products:

(11) $$C,R,G\in R^{M\times K},\;H\in R^{N_{y}\times N_{x}}.$$

Here, C={C(m,k)} is the centroid matrix, whose elements are the row-wise echo centroid column coordinates extracted from the effective streak images. For a 1024-column streak image, the centroid coordinates are within the column range of approximately 1–1024 and can be stored as a 16-bit matrix. The slant-range matrix R={R(m,k)} is obtained by combining the centroid-derived echo time, the TDC trigger-delay sequence, and the calibrated system delay. The gray-value height matrix G={G(m,k)} is generated by removing the fitted low-frequency ground trend from R and mapping the residual relative-height values to gray levels. The height mapping range is adjusted according to the target height variation; for example, mapping a 25.5m height interval to 8-bit gray values provides a height quantization interval of approximately 0.1m.

The final product H={H(x,y)} is the swath-level relative-height image after swath geometric calibration. This calibration accounts for the different sampling intervals along the cross-track scanning direction and the along-track flight direction, the spatial distribution associated with the scan-angle sequence, and the along-track displacement caused by approximately uniform forward flight. Therefore, H describes both the relative height and the local spatial distribution of wide-area ground targets within the selected scanning swath.

#### 2.2.1. Centroid Matrix Construction and Time–Angle Calibration

The first stage converts the effective streak-image sequence into a centroid matrix. For each effective streak image $I_{k}$, candidate echo spots are separated from the background using local Otsu thresholding. The local threshold at pixel (m,n) is computed within a neighborhood $N_{m,n}$:

(12) $$T_{k}\left(m,n\right)=O\left(\left\{I_{k}\left(u,v\right)|\left(u,v\right)\in N_{m,n}\right\}\right),$$

where O(·) denotes the Otsu thresholding operator. The binary candidate echo mask is then defined as

(13) $$B_{k}\left(m,n\right)=\left\{\begin{array}{cc} 1, & I_{k}\left(m,n\right)\geq T_{k}\left(m,n\right),\\ 0, & I_{k}\left(m,n\right)<T_{k}\left(m,n\right). \end{array}\right.$$

After removing small isolated regions, one-dimensional connected candidate echo components are identified along the temporal column direction:

(14) $${\left\{\Omega^{j}\left(m,k\right)\right\}}_{j=1}^{J(m,k)}=C\left(B_{k}\left(m,1:N\right)\right),$$

where C(·) denotes connected-component labeling along the column direction, $\Omega^{j}\left(m,k\right)$ is the set of temporal column indices of the *j*-th candidate echo component in row *m* and frame *k*, and J(m,k) is the number of detected components in that row.

For the *j*-th connected echo component, the intensity-weighted centroid is computed as

(15) $$C^{j}\left(m,k\right)=\frac{\sum_{n\in \Omega^{j}\left(m,k\right)}n\;I_{k}\left(m,n\right)}{\sum_{n\in \Omega^{j}\left(m,k\right)}I_{k}\left(m,n\right)}.$$

This operation provides sub-pixel localization of the echo position along the temporal sampling direction. If multiple echo components are detected in the same row, the component with the largest integrated intensity is retained:

(16) $$j^{*}\left(m,k\right)=\arg\max_{j}\left[\sum_{n\in \Omega^{j}\left(m,k\right)}I_{k}\left(m,n\right)\right].$$

The maximum-integrated-intensity criterion is used to retain the dominant effective echo for image formation. In the 532 nm ASTIL system used in this study, multiple connected components detected in the same detector row are not equivalent to physically separated first and last returns in conventional multi-return LiDAR. For building targets, the laser signal generally does not penetrate solid roofs or walls to generate meaningful roof and ground returns within the same image row. Additional components are more often associated with non-ideal spot imaging, row–column diffusion, edge scattering, or mixed echoes near structural discontinuities. Therefore, the component with the largest integrated intensity is selected as the dominant roof or ground response, while weaker diffusion-induced components are suppressed in the efficient image generation workflow. The limitation of this criterion for dense vegetation, highly reflective roof edges, and strongly layered targets is discussed later.

The selected centroid coordinate is then

(17) $$C\left(m,k\right)=C^{j^{*}\left(m,k\right)}\left(m,k\right),$$

and the centroid matrix is formed as

(18) $$C=\left\{C\left(m,k\right)\right\}\in R^{M\times K}.$$

The centroid column coordinate is further converted into calibrated echo time and spatial angle using the calibrated time–space relationship of the streak-tube detector [24]. Let

(19) q(m,k)=⌊C(m,k)⌋,b(m,k)=C(m,k)−q(m,k),

where q(m,k) is the integer column index and b(m,k) is the fractional part of the centroid coordinate. For interpolation, q(m,k) is constrained to 1≤q(m,k)≤N−1 so that the two adjacent calibration samples are valid. The calibrated echo time is obtained by linear interpolation between two adjacent temporal calibration samples:

(20) $$\Delta t_{\mathrm{echo}}\left(m,k\right)=\left[1-b(m,k)\right]M_{t}\left(m,q(m,k)\right)+b\left(m,k\right)M_{t}\left(m,q(m,k)+1\right).$$

**Figure 4:**
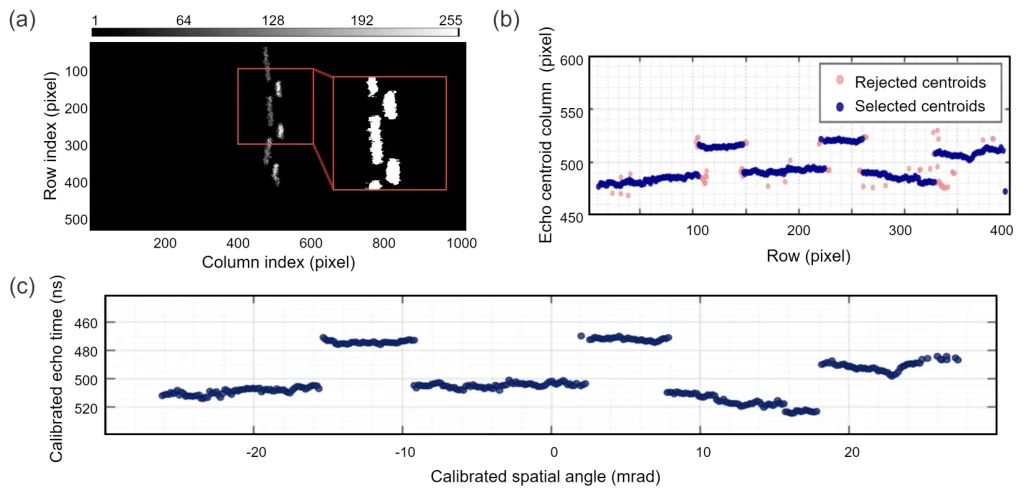
Centroid extraction and time–angle calibration of a representative ASTIL streak image. (**a**) Raw 512×1024 streak echo image, with the red inset showing a local segmented echo region used for centroid localization. (**b**) Row-wise centroid extraction and dominant-echo selection within the effective echo rows. (**c**) Calibrated echo-time and spatial-angle distribution obtained from the selected centroid coordinates using the temporal and spatial-angle calibration matrices.

Similarly, the corresponding spatial angle is interpolated as

(21) $$\theta_{\mathrm{echo}}\left(m,k\right)=\left[1-b(m,k)\right]M_{\theta }\left(m,q(m,k)\right)+b\left(m,k\right)M_{\theta }\left(m,q(m,k)+1\right),$$

where $M_{t}$ and $M_{\theta }$ are the temporal and spatial-angle calibration matrices, respectively. The calibrated echo-time information is subsequently combined with the TDC trigger-delay sequence and system delay to construct the slant-range matrix. Figure 4 provides a representative example of centroid extraction and time–angle calibration from a raw ASTIL streak image.

#### 2.2.2. Slant-Range Calibration

After centroid extraction and time–angle calibration, the centroid-derived echo time $\Delta t_{\mathrm{echo}}\left(m,k\right)$ still represents the relative echo time within the streak image of the *k*-th frame. To obtain the complete time-of-flight information, it is combined with the frame-dependent trigger-delay sequence recorded by the TDC. Specifically, $\Delta t_{\mathrm{trigger}}\left(k\right)$ provides the coarse timing offset of the *k*-th frame, while $\Delta t_{\mathrm{echo}}\left(m,k\right)$ provides the fine echo timing within the streak image. Their combination links the TDC timing sequence and the image-domain echo localization, thereby converting the centroid matrix into a slant-range matrix.

**Figure 5:**
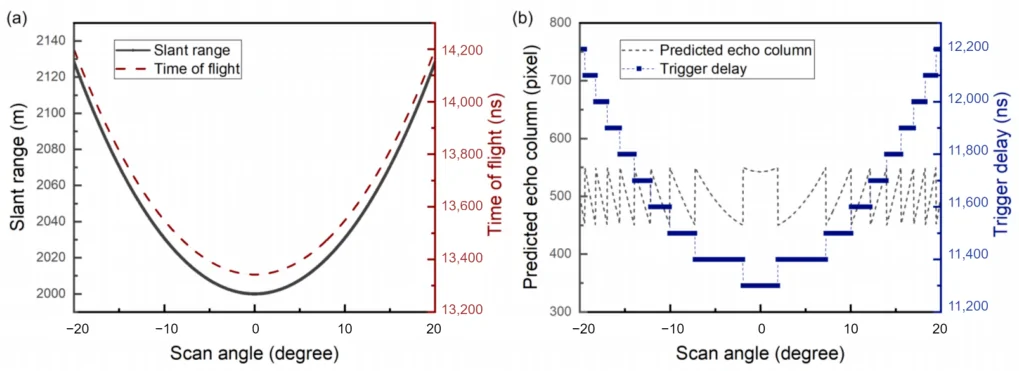
Combination of TDC trigger timing and image-domain echo timing for slant-range matrix construction. (**a**) TDC trigger-delay sequence, which provides the frame-dependent coarse timing offset for long-range echo acquisition. (**b**) Combination of the trigger delay and centroid-derived image-domain echo time for complete slant-range calculation.

The slant range is calculated by combining the interpolated image-domain echo time, the TDC trigger-delay sequence, and the calibrated system delay:

(22) $$R\left(m,k\right)=\frac{c}{2n_{\mathrm{air}}}\left[\Delta t_{\mathrm{trigger}}\left(k\right)+\Delta t_{\mathrm{echo}}\left(m,k\right)+\tau_{\mathrm{sys}}\right],$$

where *c* is the speed of light in vacuum, $n_{\mathrm{air}}$ is the refractive index of air, $\Delta t_{\mathrm{trigger}}\left(k\right)$ is the TDC-recorded trigger delay of the *k*-th frame, $\Delta t_{\mathrm{echo}}\left(m,k\right)$ is the centroid-derived echo time within the streak image, and $\tau_{\mathrm{sys}}$ is the calibrated system delay. This operation produces the slant-range matrix $R=\left\{R\left(m,k\right)\right\}\in R^{M\times K}$, which preserves the row–frame topology of C while converting each element from an image-domain centroid coordinate to a physical slant range. The calibrated spatial-angle information is retained for subsequent swath geometric calibration [23,24]. Figure 5 illustrates how the TDC trigger delay and image-domain echo timing are combined for slant-range matrix construction.

#### 2.2.3. Polynomial Relative-Height Extraction and Gray-Value Mapping

The second stage extracts height-related residual information from the slant-range matrix R. Polynomial trend fitting and ground filtering are commonly used to separate smooth terrain components from local objects in geophysical and airborne LiDAR data processing [31,32,33]. For kilometer-level ASTIL observations within a short acquisition window, the platform attitude variation is limited and the scanning geometry can be regarded as locally stable. Under this condition, a flat or slowly varying ground surface produces a smooth low-frequency trend in the slant-range sequence of each detector row. In contrast, buildings, roof boundaries, and other elevated targets appear as local deviations from this row-wise range trend. Therefore, the slant-range matrix is processed in the native row–frame sensor domain before swath geometric calibration.

In the sensor-coordinate domain, the slant range can be expressed as

(23) $$R\left(m,k\right)=R_{g}\left(m,k\right)+\Delta R_{t}\left(m,k\right)+\epsilon \left(m,k\right),$$

where $R_{g}\left(m,k\right)$ denotes the smooth ground-related range trend, $\Delta R_{t}\left(m,k\right)$ denotes the local target-related range deviation, and ϵ(m,k) represents residual measurement and modeling errors.

For each fixed detector row *m*, the slant-range values along consecutive frames form a one-dimensional range sequence:

(24) $$R_{m}\left(k\right)=R\left(m,k\right),\;k=1,\ldots ,K.$$

Since the detector row corresponds to a fixed fan-beam spatial angle, the variation in $R_{m}\left(k\right)$ is mainly caused by the frame-wise scan angle and the large-scale ground geometry. A two-stage robust row-wise fitting strategy is used. The polynomial orders are selected empirically from the present ASTIL experimental data, considering the short 0.5 s acquisition window and the smooth row-wise range trend observed in the relatively flat Dunhuang test area. First, a preliminary third-order polynomial is fitted to each row only for outlier screening. This step reduces the influence of unstable echoes, erroneous centroids, and strong local height discontinuities on the subsequent ground-trend estimation, without making the screening model overly sensitive to local building structures. The maximum number of iterations is set to 5 and the residual threshold is set to 1 m. The screened samples are excluded only from the trend estimation and are not removed from the final relative-height representation.

Second, the ground-related range trend is estimated using a fifth-order polynomial. In the tested data, this order provided a practical balance between fitting the smooth scan-related range variation and preserving local roof-height residuals. Lower-order polynomials were less flexible for the scan-related curvature, whereas higher-order fits increased the risk of absorbing local building-height variations into the estimated ground trend. The selected orders should therefore be regarded as empirical settings for the present flat-terrain, short-window dataset, rather than as universal parameters:

(25) $$P_{5,m}\left(k\right)=\sum_{i=0}^{5}a_{i,m}k^{i},$$

where $P_{5,m}\left(k\right)$ is the fitted ground-related range trend for the *m*-th detector row, and $a_{i,m}$ are the polynomial coefficients estimated from the retained slant-range samples of the same row. The maximum number of iterations is set to 5, and the residual threshold for the final fitting is set to 2 m.

The range-derived relative-height residual is then calculated as

(26) $$\Delta H\left(m,k\right)=P_{5,m}\left(k\right)-R\left(m,k\right).$$

This definition makes elevated targets tend to have positive residuals, because their measured slant ranges are generally shorter than the fitted ground-range trend. The residual suppresses the large-scale slant-range background while preserving local height-related variations associated with buildings and ground objects. Since the fitting is performed independently for each detector row, the estimation is based on slant-range samples with the same fan-beam angle, which reduces the influence of inter-row geometric differences.

For image-based representation, the residual is linearly mapped into a gray-value height matrix:

(27) $$G\left(m,k\right)=\mathrm{round}\left[\frac{\Delta H\left(m,k\right)-H_{\min}}{H_{\max}-H_{\min}}\left(L-1\right)\right],$$

where $H_{\min}$ and $H_{\max}$ define the displayed relative-height range, and L=256 for an 8-bit gray-value representation. The resulting matrix is written as

(28) $$G=\left\{G\left(m,k\right)\right\}\in R^{M\times K}.$$

Compared with the slant-range matrix R, the gray-value height matrix G has the same row–frame topology, but its elements represent gray-mapped height-related residuals rather than physical slant ranges.

This step converts the slant-range matrix into a sensor-domain gray-value relative-height matrix, providing a compact intermediate representation for subsequent swath-level relative-height image formation.

#### 2.2.4. Simplified Swath Calibration and Relative-Height Image Formation

**Figure 6:**
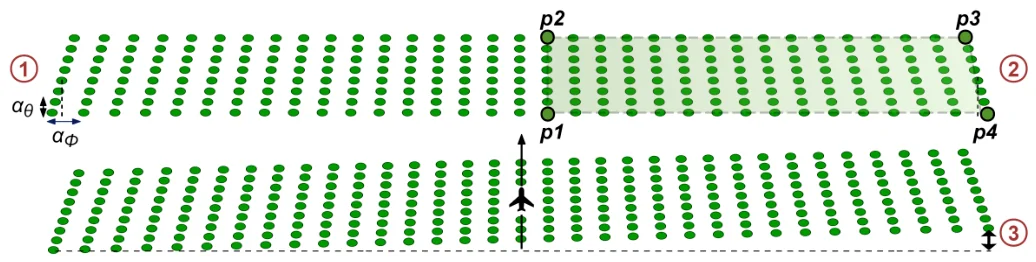
Schematic illustration of the simplified swath calibration process. Green dots represent gray-value height samples G(m,k) in the sensor-domain matrix. Step 1 performs scale correction according to the row-wise fan-beam angular resolution $\alpha_{\theta }$ and the frame-wise scan-angle interval $\alpha_{\phi }$. Step 2 applies projective correction, where the four geometry-derived swath corner points $p_{1}$–$p_{4}$ are computed from the corresponding $\theta_{m}$ and *k* values of the original matrix corners, mapping the rectangular matrix domain to a swath-shaped quadrilateral. Step 3 compensates for the along-track displacement caused by uniform forward flight, where each frame column is shifted according to $\Delta y\left(k\right)=\left(k-1\right)v_{x}/\mathrm{PRF}$. The gray-value height samples are preserved during these geometric transformations and are finally resampled onto a regular swath image grid.

This stage converts the sensor-domain gray-value height matrix $G=\left\{G\left(m,k\right)\right\}\in R^{M\times K}$ into a swath-level relative-height image H={H(x,y)}. The objective is not to obtain absolute georeferenced three-dimensional coordinates but to represent the relative height and relative spatial distribution of targets within a selected scanning swath. The geometric transformations used here are related to projective image warping and coordinate-transformation models, while rigorous airborne LiDAR mapping generally requires more complete POS/IMU-based georeferencing [25,34,35,36,37,38]. Figure 6 presents the three image-domain calibration steps used in this study, namely, scale correction, projective correction, and along-track motion compensation.

A local swath image coordinate system is defined for the final image-level representation. Its horizontal axis, $x_{\mathrm{swath}}$, corresponds to the cross-track scanning direction, and its vertical axis, $y_{\mathrm{swath}}$, corresponds to the along-track flight direction. This coordinate system is a local image coordinate system rather than a map coordinate system. Since $x_{\mathrm{LiDAR}}$ in the local LiDAR coordinate system is associated with the flight direction and $y_{\mathrm{LiDAR}}$ is associated with the cross-track scanning direction, the static coordinate relationship is written as

(29) $${\bar{x}}_{\mathrm{swath}}\left(\theta_{m},k\right)=y_{\mathrm{LiDAR}}\left(\theta_{m},k\right),\;{\bar{y}}_{\mathrm{swath}}\left(\theta_{m},k\right)=x_{\mathrm{LiDAR}}\left(\theta_{m},k\right),$$

where the overbar denotes the swath coordinates before along-track motion correction, and $\theta_{m}$ is the fan-beam spatial angle of the *m*-th detector row.

For each matrix element G(m,k), the coordinate origin is defined at the lower-left corner of the matrix. Its homogeneous matrix coordinate is

(30) $$p_{m,k}^{0}=\left[\begin{array}{c} k-1\\ m-1\\ 1 \end{array}\right],\;m=1,\ldots ,M,\;k=1,\ldots ,K.$$

The first coordinate corresponds to the frame direction, and the second coordinate corresponds to the detector-row direction. During swath calibration, only this spatial coordinate is transformed, while the gray-value height sample G(m,k) itself remains unchanged.

The simplified swath calibration is formulated as

(31) $$p_{m,k}^{c}=T_{\mathrm{motion}}\left(k\right)\;\Pi \left(T_{\mathrm{proj}}T_{\mathrm{scale}}p_{m,k}^{0}\right),$$

where $T_{\mathrm{scale}}$ is the scale correction matrix, $T_{\mathrm{proj}}$ is the projective transformation matrix, $T_{\mathrm{motion}}\left(k\right)$ is the frame-dependent along-track motion correction matrix, and Π(·) denotes homogeneous-coordinate normalization:

(32) $$\Pi \left(\left[\begin{array}{c} u\\ v\\ w \end{array}\right]\right)=\left[\begin{array}{c} u/w\\ v/w\\ 1 \end{array}\right].$$

The scale correction compensates for the difference between the row-wise angular sampling and the frame-wise scanning sampling. The row-wise angular resolution $\alpha_{\theta }$ is determined by the calibrated streak-tube detector, whereas the frame-wise scan-angle interval $\alpha_{\phi }$ is set by the scanning task. Taking the detector-row direction as the reference, the relative scale factor is approximated as

(33) $$\rho =\frac{H\alpha_{\phi }}{H\alpha_{\theta }}=\frac{\alpha_{\phi }}{\alpha_{\theta }}.$$

The scale correction matrix is therefore

(34) $$T_{\mathrm{scale}}=\left[\begin{array}{ccc} \rho & 0 & 0\\ 0 & 1 & 0\\ 0 & 0 & 1 \end{array}\right].$$

This transformation linearly stretches the frame direction relative to the detector-row direction, so that the matrix indices are expressed in a two-dimensional coordinate domain with consistent relative spatial sampling.

The projective correction maps the scaled rectangular matrix domain into the dominant swath-shaped quadrilateral determined by the scanning geometry. The four source corner points of the original matrix are defined as

(35) $$S_{0}=\left\{s_{1},s_{2},s_{3},s_{4}\right\}=\left\{\left[\begin{array}{c} 0\\ 0\\ 1 \end{array}\right],\left[\begin{array}{c} 0\\ M-1\\ 1 \end{array}\right],\left[\begin{array}{c} K-1\\ M-1\\ 1 \end{array}\right],\left[\begin{array}{c} K-1\\ 0\\ 1 \end{array}\right]\right\}.$$

After scale correction, the source corner points become

(36) $$s_{i}^{s}=T_{\mathrm{scale}}s_{i},\;i=1,\ldots ,4.$$

The corresponding target corner points are computed from the four original matrix corners using their row-wise spatial angles and frame indices:

(37) $$P_{\mathrm{swath}}=\left\{p_{1},p_{2},p_{3},p_{4}\right\},$$

where

(38) $$\begin{array}{cc} p_{1} & =q(1,1),\\ p_{2} & =q(M,1),\\ p_{3} & =q(M,K),\\ p_{4} & =q(1,K), \end{array}$$

and

(39) $$q\left(m,k\right)=\left[\begin{array}{c} {\bar{x}}_{\mathrm{swath}}\left(\theta_{m},k\right)-{\bar{x}}_{\mathrm{swath}}\left(\theta_{1},1\right)\\ {\bar{y}}_{\mathrm{swath}}\left(\theta_{m},k\right)-{\bar{y}}_{\mathrm{swath}}\left(\theta_{1},1\right)\\ 1 \end{array}\right].$$

The subtraction by the lower-left corner makes the target coordinate origin consistent with the matrix coordinate origin. The projective transformation $T_{\mathrm{proj}}$ is then estimated from the four correspondences between the scaled source corners and the geometry-derived swath corners:

(40) $$\lambda_{i}p_{i}=T_{\mathrm{proj}}s_{i}^{s},\;i=1,\ldots ,4,$$

where $\lambda_{i}$ is the homogeneous scale factor.

Within the short acquisition window, the platform motion is approximated as uniform rectilinear motion. Since the positive $y_{\mathrm{swath}}$ axis is defined along the flight direction, the along-track displacement of the *k*-th frame is

(41) $$\delta_{y}\left(k\right)=\left(k-1\right)\frac{v_{x}}{\mathrm{PRF}}=v_{x}\left(t_{k}-t_{1}\right),$$

where $v_{x}$ is the nominal flight velocity, PRF is the pulse repetition frequency, and $t_{k}$ is the acquisition time of the *k*-th frame. The corresponding motion correction matrix is

(42) $$T_{\mathrm{motion}}\left(k\right)=\left[\begin{array}{ccc} 1 & 0 & 0\\ 0 & 1 & \delta_{y}\left(k\right)\\ 0 & 0 & 1 \end{array}\right].$$

After the three coordinate transformations, the calibrated coordinate of the height sample G(m,k) is

(43) $$p_{m,k}^{c}=\left[\begin{array}{c} x_{\mathrm{swath}}\left(m,k\right)\\ y_{\mathrm{swath}}\left(m,k\right)\\ 1 \end{array}\right].$$

The relative-height image is generated by resampling the gray-value height samples onto a regular rectangular swath image grid:

(44) $$H\left(x,y\right)=W\left({\left\{G\left(m,k\right),x_{\mathrm{swath}}\left(m,k\right),y_{\mathrm{swath}}\left(m,k\right)\right\}}_{m=1,k=1}^{M,K}\right),$$

where W(·) denotes grid interpolation, image warping, or rasterization. The gray-value height samples are not modified during the geometric calibration; they are only resampled according to their calibrated spatial coordinates.

Overall, the proposed workflow converts the ASTIL raw streak-image sequence into a swath-level relative-height image through three consecutive matrix-domain steps: First, row-wise echo centroids are extracted from the raw streak images and combined with the TDC trigger timing to construct the slant-range matrix R. Second, for each detector row, the low-frequency ground-related range trend is fitted and removed from R, and the resulting height-related residuals are mapped into the gray-value height matrix G. Third, the gray-value height samples are geometrically rearranged through scale correction, projective correction, and along-track motion correction to generate the final relative-height image H in the defined local cross-track/along-track swath coordinate system. Therefore, the complete processing chain can be summarized as

(45) $${\left\{I_{k}\right\}}_{k=1}^{K}\to C\left(m,k\right)\to R\left(m,k\right)\to G\left(m,k\right)\to H\left(x,y\right).$$

This formulation exploits the row–column–frame topology of ASTIL, in which the detector row encodes fan-beam spatial sampling, the detector column encodes fine echo-time sampling, and the frame index encodes the scanning sequence. As a result, relative building height, roof structures, and height-discontinuity information can be represented as an image-level product without dense point-cloud-first reconstruction or fine POS-based point-wise georeferencing.

### 2.3. Experimental Design

#### 2.3.1. Experimental Area and Reference Data

The experimental area is located in the suburban region of Dunhuang, Gansu Province, China. The study area covers approximately 300m×600m and is characterized by relatively flat terrain, including buildings, bare ground, and locally vegetated surfaces. Three flight heights—namely, 2 km, 3 km, and 6 km above ground level—were used to evaluate the ASTIL relative-height image products at different observation scales. The three flights followed approximately the same east–west ground trajectory, so that the datasets acquired at different heights were comparable over the same geographic region.

**Figure 7:**
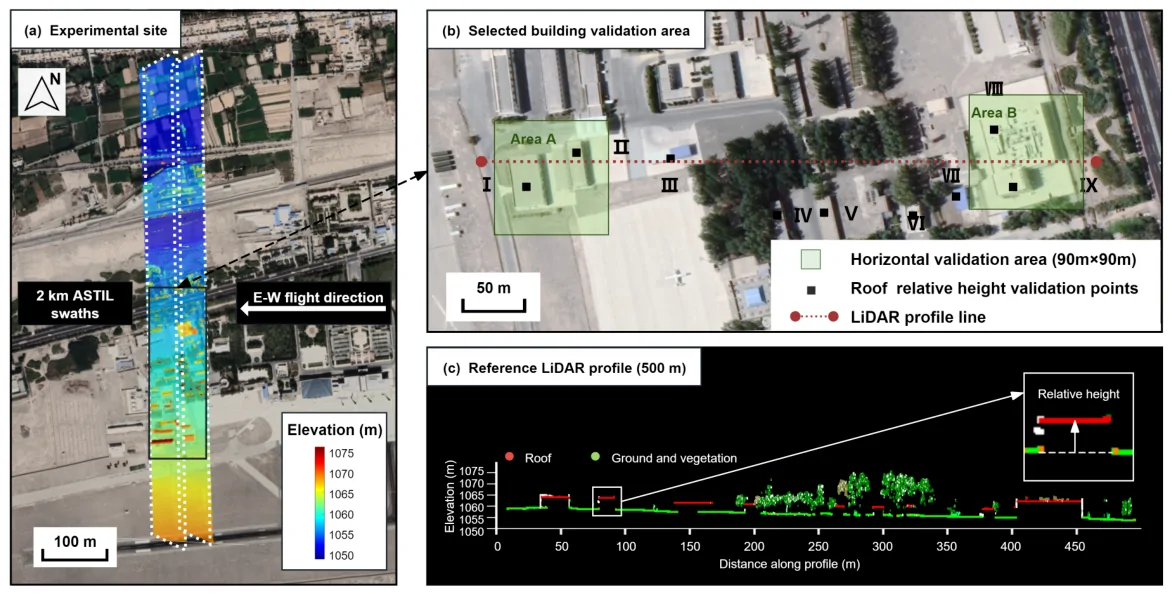
Experimental site and validation data for ASTIL observations. (**a**) Two consecutive ASTIL scanning swaths acquired at the 2 km flight height over the Dunhuang experimental area. (**b**) Selected validation samples within the approximately 300m×600m study area, including manually selected roof regions and two 90m×90m building patches. (**c**) UAV LiDAR point-cloud profile used to extract relative roof heights above the local ground surface.

Figure 7a shows two consecutive ASTIL scanning swaths acquired at the 2 km flight height. The 3 km and 6 km swaths are not separately displayed because they share the same scanning-imaging topology, with differences mainly reflected in spatial sampling resolution and ground coverage.

Figure 7b shows the selected validation samples. Nine building roof regions, labeled I–IX, were manually selected for roof-level relative-height extraction and height-consistency analysis. Two 90m×90m building patches were further selected for planar structural evaluation. The patch containing buildings I and II includes two complete rectangular buildings, while the patch containing buildings VII and IX includes composite roof structures with local small-scale roof objects. Other buildings are partially affected by vegetation or tree occlusion but still present relatively flat height distributions.

A DJI Zenmuse L1 UAV LiDAR system was used to acquire high-density reference point clouds before the ASTIL flight experiment; cross-platform LiDAR data are commonly used as reference or complementary sources for structural assessment and registration tasks [39]. According to the manufacturer’s specification, the Zenmuse L1 system can achieve a vertical accuracy of 5 cm and a horizontal accuracy of 10 cm under specified operating conditions [40]. In this study, the UAV data were collected at an altitude of approximately 100 m and had a point density of about $140\;\mathrm{points}/m^{2}$. These data were used as a high-density reference source for relative roof-to-ground height extraction rather than as an error-free ground truth. Figure 7c shows a UAV LiDAR profile of approximately 500 m in length and 1 m in width, which was used to extract the relative roof heights above the local ground surface.

#### 2.3.2. System Configuration and Data Description

The ASTIL system employs a 532 nm fan-beam laser with a nominal fan-beam spatial angle of approximately $3^{\circ }$ and a pulse repetition frequency of 2 kHz. The streak-tube imaging detector provides 512×1024 pixels, corresponding to 512 spatial pixels and 1024 temporal pixels. According to system calibration, the temporal resolution is 1.29ns/pixel, and the spatial angular resolution is 0.15mrad/pixel.

The main system parameters are listed in Table 1. The laser and streak-tube parameters define the illumination, temporal sampling, and row-wise angular sampling of the raw streak images, while the scanning-mirror parameters determine the scan-angle variation associated with the frame sequence. These parameters provide the basis for centroid extraction, range conversion, and swath geometric correction.

The nominal fan-beam spatial angle represents the main-energy coverage of the transmitted laser beam rather than a strict cutoff of the detectable echo response. With a spatial angular resolution of 0.15mrad/pixel, the measured effective echo region usually spans about 400 rows, corresponding to approximately $3.44^{\circ }$. This slightly larger image-domain support is caused by beam-edge energy falloff, target-induced spreading, receiving optical response, and streak-tube/MCP diffusion. Therefore, the effective echo rows denote the stable centroid-extraction region rather than a redefinition of the transmitted fan-beam angle. During data acquisition, the detector gain was adjusted according to the flight height to compensate for range-dependent echo attenuation, and the scanning rate was matched with the platform velocity to ensure continuous swath coverage. The imaging SNR was 25 dB, 10 dB, and 6 dB for the 2 km, 3 km, and 6 km datasets, respectively, indicating a decrease in raw echo quality with increasing ranging distance. The 6 dB case represents a low-SNR long-range observation condition. Under this condition, local thresholding and centroid localization become more sensitive to beam-edge noise, weak echo fragmentation, and local intensity fluctuations. Therefore, the 6 km results should be interpreted together with the centroid reliability, boundary sharpness, and height-error dispersion analyzed in Section 3 and Section 4.

The geometric characteristics of the ASTIL scanning swaths are summarized in Table 2. In this table, the scan direction denotes the cross-track direction perpendicular to the flight direction. The swath length and swath width describe the ground coverage of the generated relative-height images, while the point spacing is reported for three representative cases: along the flight direction, along the scan direction at the central scan angle $\phi =0^{\circ }$, and along the scan direction at the maximum scan angle $\left|\phi \right|=\phi_{\max}$.

As shown in Table 2, the swath length increases from 1456 m to 5343 m and the swath width increases from 104 m to 314 m as the ranging distance increases from 2128 m to 6568 m, indicating a larger ground coverage at longer observation distances. Such changes in sampling scale should be considered when interpreting remote-sensing image products [41,42]. The flight-direction point spacing increases from 0.30 m to 0.90 m, mainly because the ground-projected interval associated with the fixed angular sampling increases with flight height. In contrast, the scan-direction point spacing at $\phi =0^{\circ }$ changes from 0.70 m to 0.90 m, which is affected by the configured scan-angle step rather than by distance scaling alone.

The point spacing at $\left|\phi \right|=\phi_{\max}$ is used to describe the sampling variation near the swath edge. Compared with the central scan direction, the spacing difference is 0.09 m, 0.18 m, and 0.18 m for the three ranging distances, respectively. These differences are very small relative to the corresponding swath scale, with normalized magnitudes below 0.02%. Therefore, the sampling nonuniformity between the central scan region and the swath edge is considered negligible for the swath-level relative-height image generation and geometric correction in this study.

Overall, the system parameters and swath statistics define the main data conditions for the proposed relative-height image generation workflow. The row-wise angular sampling and effective echo rows determine the valid spatial extent of the centroid matrix C(m,k); the temporal resolution supports the conversion to the slant-range matrix R(m,k); and the scan-angle step, swath coverage, and point-spacing characteristics constrain the resampling from G(m,k) to the final relative-height image H(x,y).

## 3. Results

The results are organized into two parts: The first part examines the intermediate and final image products generated by the proposed ASTIL processing workflow, including slant-range matrix construction, relative-height image generation, and image-level coverage and data-volume analysis. The second part evaluates the generated products at the building scale by comparing them with optical images, UAV LiDAR reference data, and calibrated ASTIL point-cloud projections, followed by building-boundary and relative roof-height assessments.

### 3.1. Algorithm Processing Results and Image-Level Analysis

This subsection focuses on the image generation process. The raw ASTIL streak-image sequences are first converted into centroid and slant-range matrices. The slant-range matrices are then transformed into relative-height images through ground-trend removal, height mapping, and scan-geometry calibration. Finally, image-level indicators are used to summarize the effective imaging area, acquisition efficiency, and output data volume of the generated products.

#### 3.1.1. Raw Echo Processing and Slant-Range Matrix Construction

**Figure 8:**
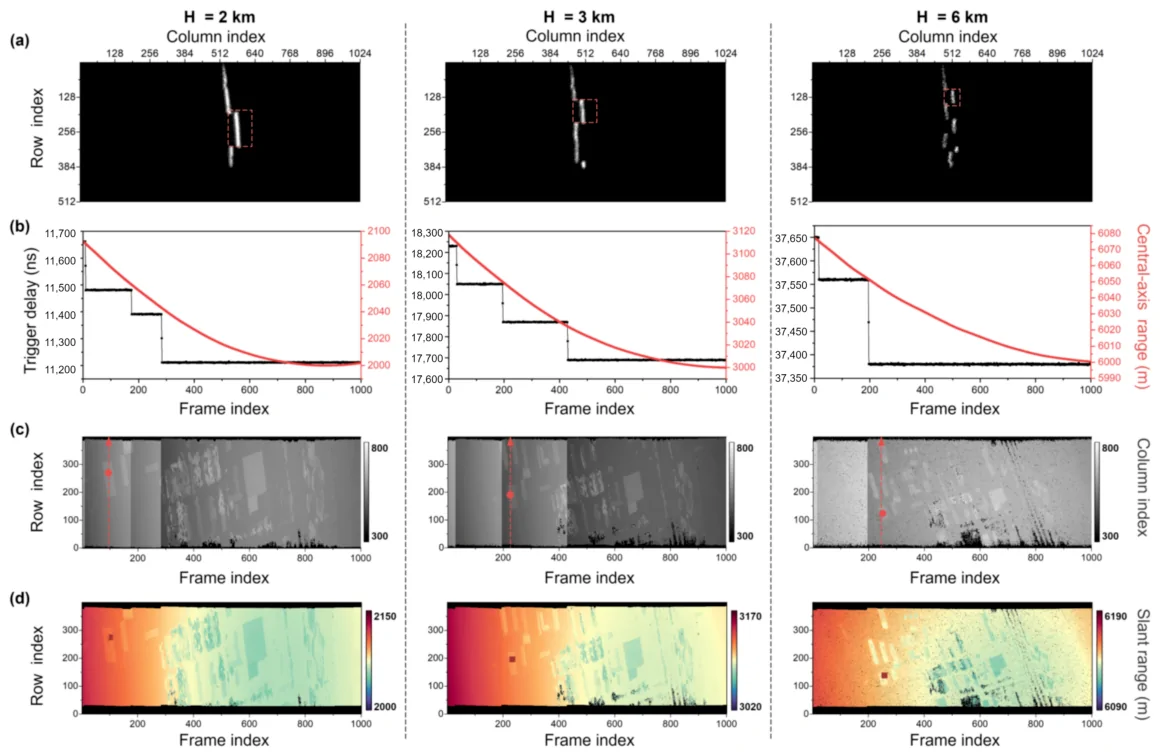
Intermediate results of ASTIL slant-range matrix construction at three flight heights. Columns correspond to the 2 km, 3 km, and 6 km flight heights, respectively. (**a**) Representative raw streak images, where the red dashed boxes indicate corresponding roof echo regions. (**b**) Dynamic trigger-delay control and the corresponding central-axis reference range $R_{0}\left(k\right)$ at $\theta =0^{\circ }$ within the selected 0.5 s acquisition windows. (**c**) Centroid column matrices C(m,k) extracted from 1000 consecutive streak images. The red dashed vertical lines indicate the frame positions associated with the roof echoes in (**a**), and the red dots denote the extracted centroid coordinates of the marked roof targets. (**d**) Slant-range matrices R(m,k) converted from centroid coordinates using temporal calibration, trigger delay, and system-delay correction. The red dots mark the corresponding roof positions after range conversion.

Figure 8 shows the intermediate results of slant-range matrix construction at the 2 km, 3 km, and 6 km flight heights. The columns correspond to the three flight heights, and the four rows show the main processing stages from raw streak imaging to slant-range matrix generation.

Figure 8a presents representative raw streak images. The red dashed boxes indicate corresponding roof echo regions under different flight heights. Owing to differences in flight height, scan angle, detector gain, and imaging SNR, the roof echoes show different spatial extents along the detector-row direction. The detector-column direction is governed by the streak-tube temporal sampling, so the column displacement between roof and ground echoes mainly reflects their relative range difference. At longer ranges, however, reduced SNR, atmospheric propagation effects, and centroid-localization uncertainty may affect the extracted echo positions.

Figure 8b shows the dynamic trigger-delay control within the selected 0.5 s acquisition windows. Since the system operates at a pulse repetition frequency of 2 kHz, each window contains approximately 1000 consecutive streak images. The trigger delay is adjusted in discrete steps to keep the effective echoes within the temporal sampling range of the streak-tube detector. The red curve represents the central-axis reference range $R_{0}\left(k\right)$ at $\theta =0^{\circ }$, which is determined by the trigger-delay setting. This reference range is used for temporal-window control and does not represent the full fan-beam slant-range distribution.

Figure 8c shows the centroid column matrices C(m,k) extracted from the raw streak-image sequences. Under dynamic trigger-delay control, most valid echo centroids are maintained within approximately 300–800 columns. The abrupt column shifts correspond to the stepwise trigger-delay adjustment. With a temporal resolution of 1.29ns/pixel, a trigger-delay adjustment of 100 ns corresponds to approximately 77 pixels, while a 200 ns adjustment corresponds to approximately 155 pixels. These shifts keep the echoes near the central column region. Missing centroids near the low-row edge are mainly caused by weak beam-edge echoes whose intensities are close to the background noise level.

Figure 8d shows the slant-range matrices R(m,k) obtained from centroid coordinates, temporal calibration, trigger delay, and system-delay correction. Compared with $R_{0}\left(k\right)$ in Figure 8b, R(m,k) provides a two-dimensional range distribution over detector row *m* and frame index *k*, including the range variation associated with different fan-beam spatial angles. The matrices preserve the continuous range variation across the scanning sequence and provide the basis for subsequent ground-trend removal, gray-value height mapping, and swath-level image generation. Because the system delay was determined from laboratory calibration and was not further refined using ground control points, the absolute range may contain a systematic offset. This offset is approximately constant within each acquisition window and has limited influence on the relative-height extraction.

#### 3.1.2. Relative-Height Image Generation at Multiple Flight Heights

**Figure 9:**
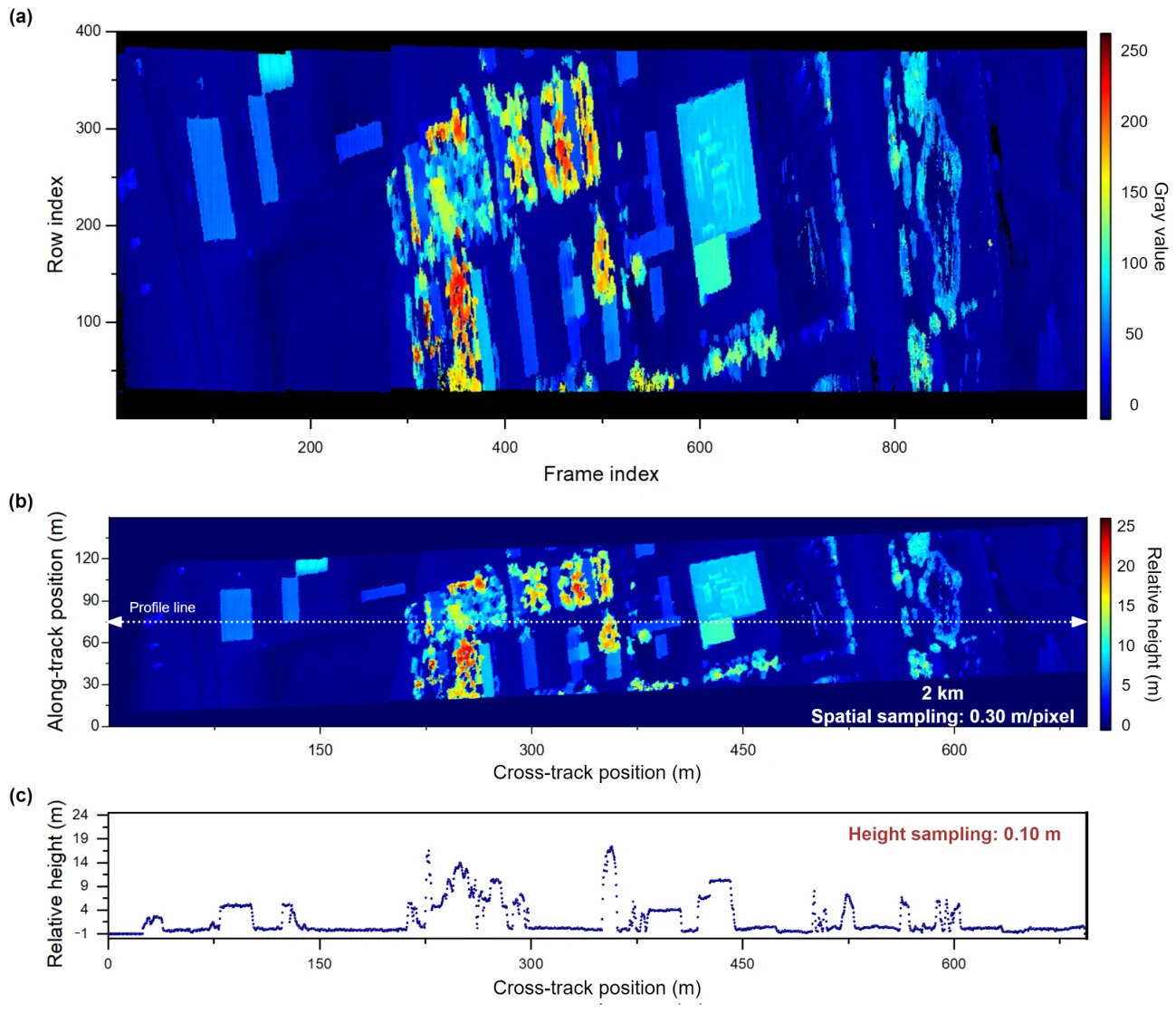
Relative-height image generation result for the 2 km ASTIL dataset. (**a**) Sensor-domain relative-height matrix obtained from the 400×1000 slant-range matrix after row-wise ground-trend removal and residual extraction. (**b**) Cropped effective relative-height image after scan-geometry calibration, along-track flight-motion compensation, and interpolation-based resampling, with an image size of 500×2313 pixels and a spatial sampling interval of 0.30 m. (**c**) Relative-height profile extracted along the profile line marked in (**b**). The height sampling interval is 0.1 m.

**Figure 10:**
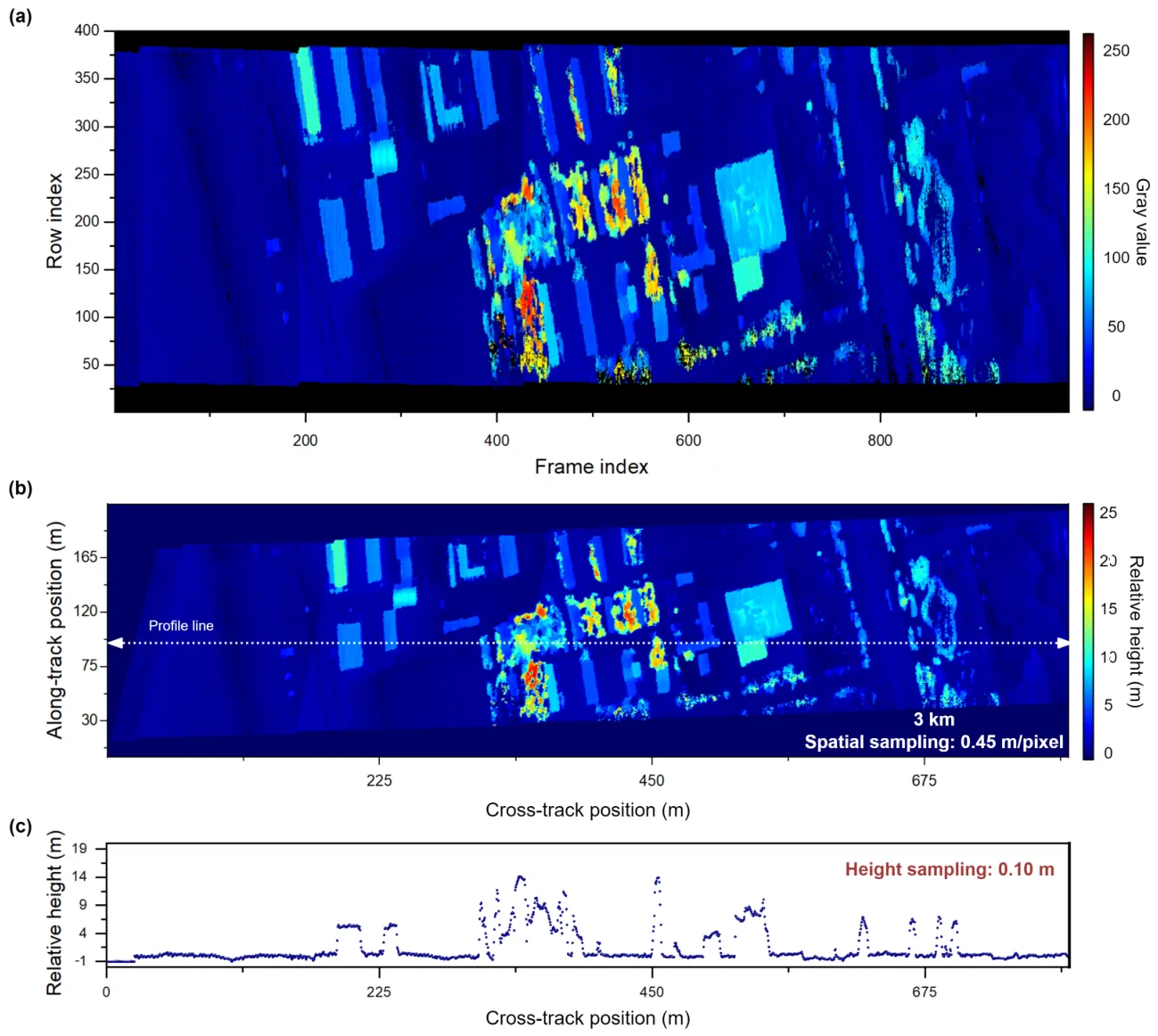
Relative-height image generation result for the 3 km ASTIL dataset. (**a**) Sensor-domain relative-height matrix obtained from the 400×1000 slant-range matrix after row-wise ground-trend removal and residual extraction. (**b**) Cropped effective relative-height image after scan-geometry calibration, along-track flight-motion compensation, and interpolation-based resampling, with an image size of 473×1764 pixels and a spatial sampling interval of 0.45 m. (**c**) Relative-height profile extracted along the profile line marked in (**b**). The height sampling interval is 0.1 m.

**Figure 11:**
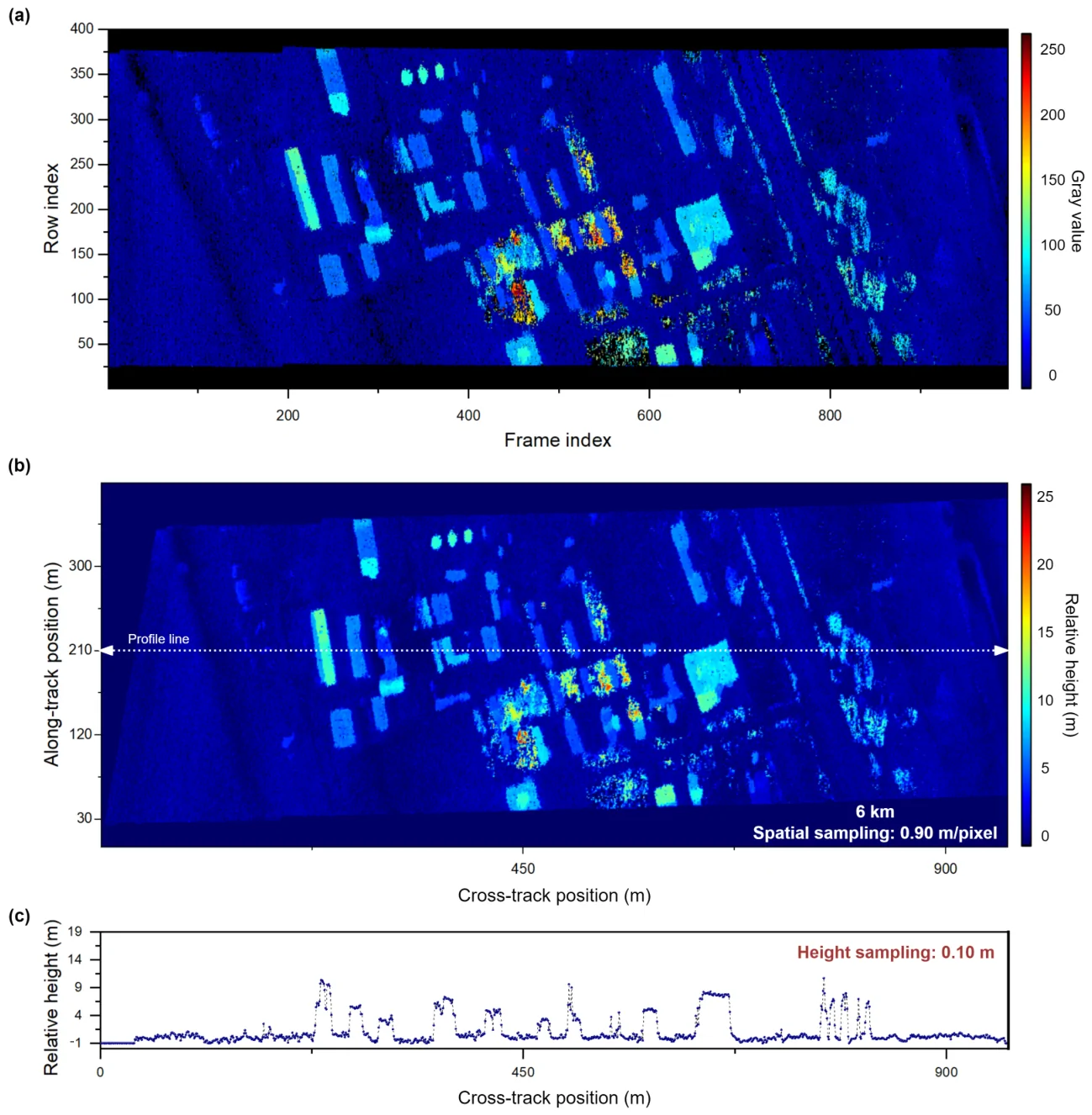
Relative-height image generation result for the 6 km ASTIL dataset. (**a**) Sensor-domain relative-height matrix obtained from the 400×1000 slant-range matrix after row-wise ground-trend removal and residual extraction. (**b**) Cropped effective relative-height image after scan-geometry calibration, along-track flight-motion compensation, and interpolation-based resampling, with an image size of 433×1073 pixels and a spatial sampling interval of 0.90 m. (**c**) Relative-height profile extracted along the profile line marked in (**b**). The height sampling interval is 0.1 m.

Figure 9, Figure 10 and Figure 11 present the relative-height image generation results at the 2 km, 3 km, and 6 km flight heights. For each height, three products are shown: the sensor-domain relative-height matrix, the scan-geometry-calibrated relative-height image, and the extracted relative-height profile.

For all three datasets, the input slant-range matrix has a size of 400×1000, corresponding to the effective echo rows and the selected 0.5 s acquisition window. Based on the row-wise ground-trend removal described in Section 2, the slowly varying range component caused by ground topography, scan geometry, and platform motion is suppressed. The residuals are then converted into sensor-domain relative-height matrices, as shown in Figure 9a, Figure 10a and Figure 11a.

The relative-height values are mapped to 8-bit gray values using a 25.5 m height interval in the target area, corresponding to a height sampling interval of approximately 0.1 m per gray level. The same height sampling interval is used for all three flight heights, ensuring a consistent gray-value scale for roof-to-ground height representation.

After scan-geometry calibration and image resampling, the sensor-domain matrices are transformed into swath-level relative-height images in the local cross-track/along-track coordinate system. The image sizes reported in Figure 9, Figure 10 and Figure 11 correspond to cropped effective display regions after along-track flight-motion compensation and interpolation-based resampling, rather than to the full theoretical swath extents listed in Table 2. For the 2 km dataset, the 400×1000 matrix is calibrated and cropped into a 500×2313 pixel image with a spatial sampling interval of 0.30 m. For the 3 km dataset, the calibrated and cropped image size is 473×1764 pixels, with a spatial sampling interval of 0.45 m. For the 6 km dataset, the calibrated and cropped image size is 433×1073 pixels, with a spatial sampling interval of 0.90 m. These results show the expected scale change with flight height: the spatial sampling interval becomes coarser as the flight height increases.

The extracted profiles further show the vertical transition between roof and surrounding ground in the generated relative-height images. Therefore, this stage verifies the conversion from raw ASTIL measurements to calibrated relative-height image products at different flight heights.

#### 3.1.3. Image-Level Coverage Efficiency and Data-Volume Analysis

To evaluate the generated relative-height products at the image level, two indicators are used: image acquisition efficiency and output-to-raw data volume ratio. The effective image area is estimated as

(46) $$A_{\mathrm{img}}\approx N_{f}N_{r,\mathrm{eff}}\Delta_{s}^{2},$$

where $N_{f}=1000$ is the number of frames within the selected 0.5 s acquisition window, $N_{r,\mathrm{eff}}=350$ is the effective row number used for stable area estimation, and $\Delta_{s}$ is the spatial sampling interval of the generated relative-height image. The value of 350 is selected from the 400 effective echo rows by excluding the low-SNR beam-edge region, which may affect the robustness of ground-trend fitting and residual extraction.

The image acquisition efficiency is defined as

(47) $$E_{\mathrm{img}}=\frac{A_{\mathrm{img}}}{T_{\mathrm{acq}}},$$

where $T_{\mathrm{acq}}=0.5\;s$ is the acquisition duration. The output-to-raw data volume ratio is defined as

(48) $$\eta_{\mathrm{prod}}=\frac{D_{\mathrm{prod}}}{D_{\mathrm{raw}}}\times 100\%,$$

where $D_{\mathrm{prod}}$ and $D_{\mathrm{raw}}$ denote the data volumes of the generated relative-height image and the original raw streak-image sequence, respectively. Since both are represented as 8-bit gray-scale images, the data volume is proportional to the number of pixels.

Table 3 summarizes the image-level coverage and data-volume indicators of the generated relative-height products. Within the same 0.5 s acquisition window, the estimated image area increases from 31,500 $m^{2}$ at 2 km to 70,875 $m^{2}$ at 3 km and 283,500 $m^{2}$ at 6 km. The corresponding image acquisition efficiency increases from 63,000 $m^{2}/s$ to 141,750 $m^{2}/s$ and 567,000 $m^{2}/s$. This increase mainly reflects the geometric enlargement of the ground footprint at higher flight heights, rather than a monotonic improvement in imaging quality. Therefore, $A_{\mathrm{img}}$ and $E_{\mathrm{img}}$ should be interpreted jointly with the coarser spatial sampling, lower raw echo SNR, and larger height-error dispersion observed at longer ranges.

The output-to-raw data volume ratio is 0.221%, 0.159%, and 0.089% for the 2 km, 3 km, and 6 km datasets, respectively. Since the three datasets have the same raw input size of 1000×512×1024 pixels, the lower ratio at higher flight heights is mainly associated with the smaller number of pixels in the calibrated and cropped output images. The output image size in Table 3 therefore refers to the effective display region used in Figure 9, Figure 10 and Figure 11, while the swath length and swath width in Table 2 describe the nominal geometric coverage of the scanning swath. This ratio should be regarded as an indicator of the compact image-domain product generated for rapid preview, preliminary mapping, and reduced data transfer, rather than as a measure of lossless compression or reconstruction accuracy. Together, these indicators describe the generated relative-height products in terms of effective imaging area, acquisition efficiency, spatial sampling, and output data volume.

### 3.2. Building-Scale Feature Analysis and Evaluation

This subsection evaluates whether the generated relative-height images can support building-scale interpretation. The proposed image products are compared with optical image patches, UAV LiDAR reference data, and calibrated ASTIL point-cloud projections. Building-boundary preservation is then assessed using gradient-derived edge responses and UAV-derived reference boundaries, with flight-stability data used to interpret possible geometric deviations. Finally, relative roof-height errors are evaluated for selected flat-roof targets.

#### 3.2.1. Comparison with Reference Remote-Sensing Products

**Figure 12:**
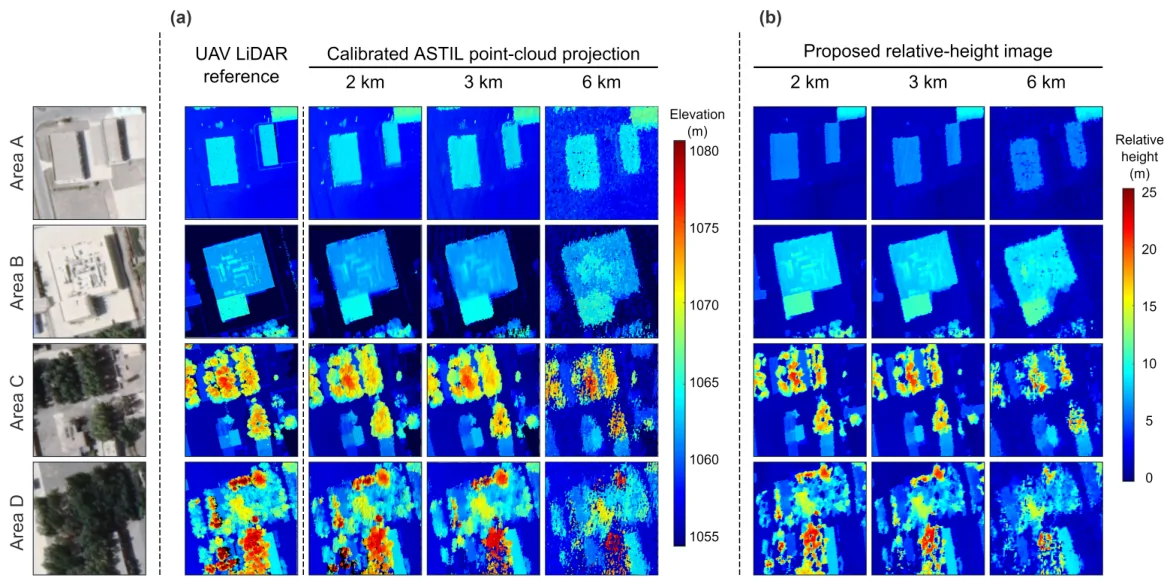
Comparison of calibrated ASTIL point-cloud projections and proposed relative-height images for four 90m×90m building validation areas. The left column shows the optical image patches of Areas A–D. (**a**) UAV LiDAR reference data acquired at approximately 100 m flight height and calibrated ASTIL point-cloud projections generated from the 2 km, 3 km, and 6 km datasets. (**b**) Relative-height images generated by the proposed method from the raw ASTIL echo data at the corresponding flight heights. The color scales represent elevation for the point-cloud projections and relative height for the proposed image products.

Figure 12 compares optical image patches, UAV LiDAR reference data, calibrated ASTIL point-cloud projections, and the proposed relative-height images for four 90m×90m validation areas. The optical images provide surface texture information, but building boundaries are not always distinct. In Areas A and B, roof surfaces have similar brightness and texture to the surrounding ground. In Areas C and D, trees and vegetation partially obscure the buildings. In contrast, LiDAR-based products directly describe elevation distribution and are less affected by surface color and texture, allowing roof-height discontinuities to be identified where valid laser returns are available.

In Figure 12a, the UAV LiDAR reference data provide the most detailed roof geometry owing to low-altitude acquisition, smaller ground footprint, higher point density, and higher elevation accuracy. Small rooftop structures in Area B are clearly represented in the UAV point cloud. In the ASTIL point-cloud projections, these fine structures become less distinct as the flight height increases from 2 km to 6 km. This degradation is mainly caused by the enlarged ground sampling interval and the increased projected footprint width of the fixed-angular fan-beam illumination at longer ranges. For the 6 km dataset, reduced raw echo SNR caused by atmospheric transmission loss and laser-energy attenuation further decreases centroid-extraction stability, leading to more noisy points and fewer reliable returns.

Figure 12b presents the relative-height images generated from the raw ASTIL echo data. Unlike elevation-based point-cloud projections, the proposed images represent height differences relative to the local ground trend. After scan-geometry calibration under the nominal level-flight imaging model, the sensor-domain relative-height matrices are transformed into two-dimensional image products in the local swath coordinate system.

For Areas A and B, the proposed relative-height images show stronger local height contrast than the calibrated ASTIL point-cloud projections because the large-scale ground elevation component is suppressed during row-wise trend removal. For Areas C and D, where vegetation introduces occlusion and scattered returns, dominant-echo selection in the raw waveform channel reduces part of the weak and diffuse vegetation-related responses before image formation. As a result, the relative-height images present more continuous height patterns for the dominant roof and ground surfaces where valid returns are available.

#### 3.2.2. Building-Boundary Preservation and Flight-Stability Analysis

**Figure 13:**
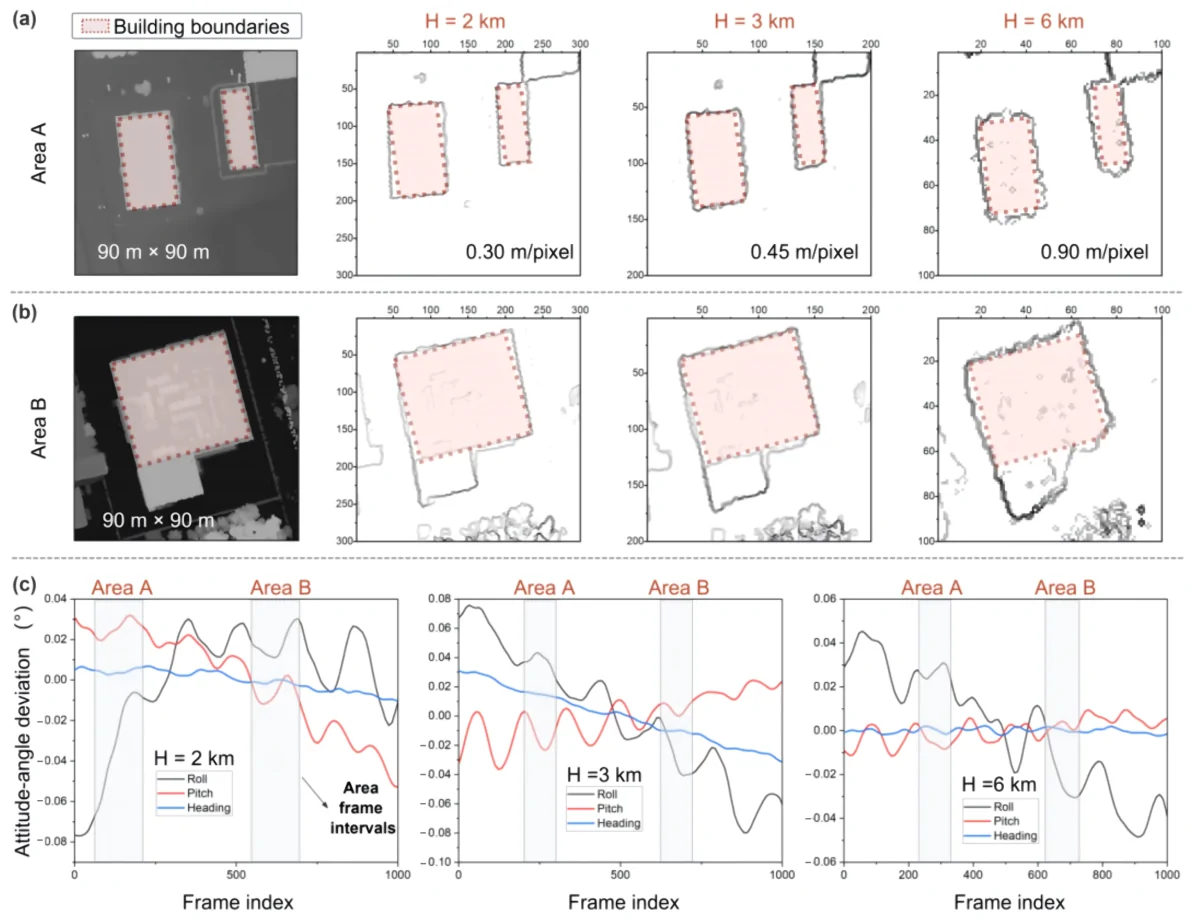
Building-boundary validation of the proposed relative-height images and flight-attitude variations during the selected ASTIL acquisition windows. (**a**) Boundary comparison for Area A. The left panel shows the 90m×90m validation area with manually delineated building boundaries, and the following panels show the gradient-derived edge responses from the proposed relative-height images at 2 km, 3 km, and 6 km flight heights. (**b**) Boundary comparison for Area B. The red dashed polygons indicate the UAV-derived reference building boundaries. The spatial sampling intervals of the relative-height images are 0.30, 0.45, and 0.90 m/pixel for the 2 km, 3 km, and 6 km datasets, respectively. (**c**) Roll, pitch, and heading variations relative to the mean attitude within the 0.5 s acquisition windows. The gray shaded regions indicate the frame intervals corresponding to Areas A and B.

To evaluate the building-scale geometric representation of the proposed relative-height images, two 90m×90m validation areas were selected, as shown in Figure 13a,b. For the 2 km, 3 km, and 6 km datasets, the spatial sampling intervals are 0.30, 0.45, and 0.90 m/pixel, respectively. Therefore, the same 90m×90m area corresponds to approximately 300×300, 200×200, and 100×100 pixels at the three flight heights.

Building edge responses were extracted from the proposed relative-height images using gradient information and compared with manually delineated building boundaries derived from the UAV LiDAR reference data. Gradient-based edge detection is commonly used to characterize local image structures and boundary responses [43]. As shown in Figure 13a,b, the extracted edges are generally consistent with the reference boundaries in footprint size and orientation. This indicates that the proposed method preserves the main building geometry in the image domain, although the images are generated using nominal scan-geometry calibration rather than full POS/IMU-based point-cloud georeferencing.

The boundary responses become coarser with increasing flight height. This behavior is consistent with the increase in ground sampling interval from 0.30 m/pixel at 2 km to 0.90 m/pixel at 6 km. In addition, the projected footprint width of the fixed-angular fan-beam illumination increases with propagation distance, causing stronger spatial averaging near roof boundaries. Therefore, reduced boundary sharpness at longer ranges is mainly related to coarser spatial sampling, enlarged projected footprint, and lower raw echo SNR.

The proposed geometric correction is based on short-term level-flight and uniform-motion assumptions within each 0.5 s acquisition window. To examine these assumptions, flight attitude and position stability were analyzed. Table 4 summarizes the altitude and attitude standard deviations, together with the along-track displacement for each flight height. The along-track displacements are 28.46 m, 29.44 m, and 27.00 m for the 2 km, 3 km, and 6 km datasets, respectively, compared with the nominal displacement of approximately 30 m obtained from the assumed flight speed of 60m/s.

Figure 13c further shows the roll, pitch, and heading variations relative to the mean attitude within each 0.5 s window. The gray shaded regions indicate the frame intervals corresponding to Areas A and B. Although the overall attitude variation is small, local roll and pitch fluctuations remain within the building imaging intervals. These variations may introduce small boundary shifts, rotations, or scale differences. In addition, deviations from the nominal along-track displacement may cause local along-track scale errors in the output images. In the present experiments, the roll and pitch standard deviations remain below 0.035°, the altitude standard deviation is below 0.034 m, and the along-track displacement differs from the nominal 30 m by less than approximately 3 m within each 0.5 s window. These values are used as empirical validity conditions for the simplified calibration in this dataset, rather than as universal thresholds. Therefore, residual mismatch between the gradient-derived edge responses and the UAV-derived reference boundaries should be interpreted as the combined effect of spatial sampling, beam-footprint enlargement, echo SNR degradation, attitude variation, platform-speed fluctuation, and simplified flight-motion correction.

#### 3.2.3. Relative Roof-Height Accuracy Evaluation

To further evaluate the height representation capability of the proposed relative-height images, nine flat-roof building targets were selected within the study area. The reference roof-to-ground heights were obtained from UAV LiDAR point-cloud profiles, as illustrated in Figure 7c. For each target, the roof surface and adjacent local ground surface were manually identified from the UAV profile, and their height difference was used as the reference relative roof height.

For the ASTIL-derived relative-height images, roof heights were obtained by selecting pixels corresponding to each building roof and converting their gray values into relative-height values. The relative-height image was quantized with a height sampling interval of 0.1 m. In the gray-value mapping, a gray value of 0 corresponds to −1m, so that slightly depressed ground regions below the fitted local ground trend can also be represented.

**Figure 14:**
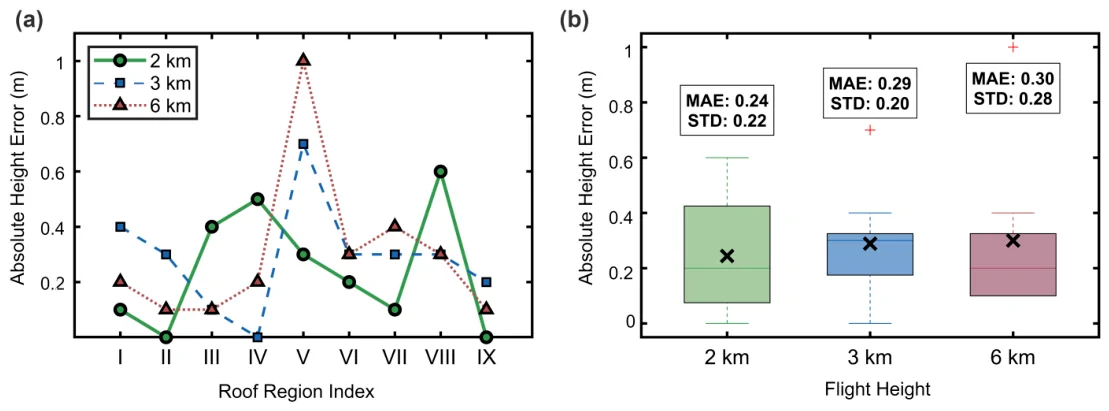
Relative roof-height error evaluation for nine flat-roof building targets. (**a**) Absolute height errors of individual targets at the 2 km, 3 km, and 6 km flight heights. The reference roof-to-ground heights were measured from UAV LiDAR point-cloud profiles. (**b**) Statistical distributions of the absolute height errors. The annotations indicate the mean absolute error (MAE) and standard deviation (STD) for each flight height.

Figure 14a shows the absolute height errors of the nine flat-roof targets at the 2 km, 3 km, and 6 km flight heights. Most targets exhibit sub-meter errors, indicating that the proposed relative-height images preserve the main roof-to-ground height information over the tested altitude range. For the 3 km and 6 km datasets, several targets show larger individual errors, suggesting that height estimation is affected by local roof selection, edge-pixel mixing, ground-trend fitting uncertainty, and echo quality in addition to flight height.

Figure 14b summarizes the statistical distributions of the absolute height errors. The mean absolute errors are 0.24 m, 0.29 m, and 0.30 m for the 2 km, 3 km, and 6 km datasets, respectively. The corresponding standard deviations are 0.22 m, 0.20 m, and 0.28 m. The mean error remains close to 0.3 m for all three flight heights, showing that the proposed method maintains a comparable relative-height estimation level from 2 km to 6 km. The larger dispersion at 6 km indicates reduced stability for some targets, which is consistent with the coarser spatial sampling interval, increased projected footprint width, and lower raw echo SNR at longer range. The nine flat-roof targets provide a preliminary building-level validation, but they are not sufficient for a statistically exhaustive assessment over all roof types, terrain conditions, and target materials.

## 4. Discussion

The experimental results demonstrate that the row–column–frame topology of ASTIL raw streak images can be transformed into an image-domain representation of relative building height. The intermediate products in Figure 8 show that the centroid matrices and slant-range matrices preserve the detector-row and frame-index organization of the raw data. This is consistent with the recent trend of using LiDAR image representations, including range-view and projection-based representations, to improve the computational efficiency of point-cloud processing [15,16,17,18]. Unlike point-cloud-to-image projection, the proposed workflow generates the relative-height image directly from the native ASTIL sensor topology. Therefore, the method is better interpreted as a topology-driven image-formation strategy for rapid preliminary mapping, rather than as a replacement for rigorous point-cloud reconstruction.

The absolute slant range may contain a systematic offset because the system delay was calibrated in the laboratory and was not further refined using ground control points. A constant delay error Δτ introduces an approximately constant range bias of $c\Delta \tau /(2n_{\mathrm{air}})$. In the proposed relative-height extraction, such a constant bias is largely absorbed by the row-wise polynomial ground-trend fitting, so the residual $\Delta H\left(m,k\right)=P_{5,m}\left(k\right)-R\left(m,k\right)$ is mainly affected by spatially or temporally varying delay errors rather than by a constant delay offset. Nevertheless, if the delay error varies with detector row, frame index, temperature, or acquisition time, it may introduce residual height distortions. Future work should therefore include in-scene calibration targets or ground control constraints to quantify and compensate for delay drift.

The multi-altitude results reveal a clear trade-off between coverage and local image detail. Within the same 0.5 s acquisition window, the estimated image area increases from 31,500 $m^{2}$ at 2 km to 283,500 $m^{2}$ at 6 km, whereas the spatial sampling interval changes from 0.30 m to 0.90 m. Thus, the higher-altitude datasets provide larger coverage but coarser building-boundary representation. This should not be interpreted as a simple improvement in system performance. The larger coverage is mainly a geometric consequence of increased flight height, while the 6 km data also have lower raw echo SNR, an enlarged projected footprint, and larger height-error dispersion. Recent airborne LiDAR building-contour and building-reconstruction studies have also shown that roof-contour extraction and building reconstruction are affected by contour complexity, nonuniform point density, occlusion, and incomplete coverage [8,9]. These findings support the interpretation that the reduced boundary sharpness in the 6 km relative-height images is mainly related to coarser spatial sampling, footprint enlargement, and lower echo reliability at longer ranges.

The building-scale comparisons indicate that the proposed relative-height images and calibrated ASTIL point-cloud projections provide different but complementary representations. The UAV LiDAR data preserve the most detailed roof geometry because of low-altitude acquisition, higher point density, and higher elevation accuracy. Compared with calibrated ASTIL point-cloud projections, the proposed relative-height images suppress the large-scale ground elevation component through row-wise trend removal and enhance local roof-to-ground height contrast. Recent building-extraction studies have emphasized that combining LiDAR point clouds and remote-sensing images can help reduce the influence of optical texture, shadow, and occlusion in large-scale building extraction [6]. In this study, the proposed product does not fuse optical imagery, but it follows a similar motivation: using height-related LiDAR information to provide a more stable building-structure representation than texture-only images.

The dominant-echo selection strategy is another practical assumption of the proposed workflow. The maximum-integrated-intensity criterion is suitable for efficient image generation in the tested building scenes because the additional components detected in one detector row are usually associated with spot diffusion, mixed echoes, or edge scattering rather than with physically meaningful first/last returns through the building target. This differs from conventional multi-return LiDAR, where chronological return order can be used to interpret canopy and terrain responses. However, the criterion may be less reliable in dense vegetation, highly reflective roof edges, or strongly layered structures. More accurate mixed-echo identification and filtering will be required when the method is extended to scenes with complex vegetation or multi-layer targets.

The polynomial ground-trend fitting also has a limited validity range. The third-order outlier screening and fifth-order trend fitting were selected empirically according to the present short-window ASTIL data and the relatively flat Dunhuang test area. For areas with stronger terrain relief, steep slopes, or more complex ground structures, the smooth row-wise trend assumption may no longer hold. In such cases, terrain variations may be mixed with building-height residuals, producing biased relative-height images or geometric artifacts. Therefore, the current method is most suitable for relatively flat urban or suburban scenes within short, stable acquisition windows. Future work should investigate adaptive polynomial-order selection, DEM-assisted terrain compensation, and more robust ground-trend estimation for non-flat topographies.

The geometric consistency of the generated images is limited by the simplified scan-geometry calibration. The attitude and displacement results in Table 4 and Figure 13c show that the selected 0.5 s windows satisfy the short-term stable-flight assumption at the tested scale, with roll and pitch standard deviations below 0.035° and along-track displacement deviations below approximately 3 m from the nominal displacement. These values provide empirical validity conditions for the present dataset, rather than universal thresholds. Larger attitude variations, stronger speed fluctuations, or longer acquisition windows would require more complete POS/IMU-based correction.

The roof-height evaluation further confirms the applicability and limitations of the relative-height image product. The mean absolute errors of the nine flat-roof targets are 0.24 m, 0.29 m, and 0.30 m at 2 km, 3 km, and 6 km, respectively, indicating decimeter-level roof-to-ground height consistency under lightweight scan-geometry calibration. The larger dispersion at 6 km is consistent with the coarser sampling interval, increased projected footprint width, and lower raw echo SNR. However, the nine flat-roof targets provide only a preliminary validation and do not cover all roof shapes, materials, vegetation conditions, or terrain types. Therefore, the reported errors should be interpreted as building-scale evidence for the tested scene rather than as a universal accuracy guarantee.

Overall, the proposed method provides a compact image-domain representation for rapid wide-area building-structure mapping, rather than a replacement for high-precision point-cloud reconstruction or rigorous POS/IMU-based georeferencing. The output-to-raw data volume ratio below 0.3% should be interpreted as the data-volume compactness of the generated cropped image products, not as a measure of lossless compression or reconstruction accuracy. This compact product is useful for rapid preview, preliminary structural interpretation, and reduced data transfer, while the full raw sequence or rigorously reconstructed point cloud remains necessary for high-precision surveying. Future work should focus on adaptive trend modeling, mixed-echo filtering, improved geometric correction, low-SNR robustness, and multi-swath image mosaicking [44,45].

## 5. Conclusions

This study proposes a topology-driven relative-height image generation method for long-range ASTIL. By exploiting the intrinsic row–column–frame structure of raw streak images, the method constructs centroid, slant-range, and gray-value relative-height matrices and converts them into scan-geometry-calibrated swath-level relative-height images. The workflow provides a compact image-domain representation for preliminary wide-area building-structure mapping without dense point-cloud-first reconstruction or rigorous point-wise POS/IMU trajectory correction.

Airborne experiments at 2 km, 3 km, and 6 km verified the feasibility of the proposed method. Within a 0.5 s acquisition window, the generated relative-height images achieved spatial sampling intervals of 0.30 m, 0.45 m, and 0.90 m, respectively. The estimated effective imaging area increased from 31,500 m^2^ to 283,500 m^2^, while the cropped image products occupied less than 0.3% of the raw streak-image sequence volume. Building-scale evaluation showed that the generated images preserved major building footprints, roof-height discontinuities, and boundary orientations. For nine flat-roof targets, the mean absolute roof-to-ground height errors were 0.24 m, 0.29 m, and 0.30 m at 2 km, 3 km, and 6 km, respectively.

The method also has limitations. Larger coverage at higher flight heights is accompanied by coarser spatial sampling, lower raw echo SNR, enlarged projected footprints, and reduced boundary sharpness. The simplified calibration and empirical polynomial trend model are most suitable for short, stable acquisition windows over relatively flat terrain. Future work will focus on adaptive ground-trend modeling, mixed-echo filtering, POS/IMU-assisted geometric correction, low-SNR enhancement, and multi-swath relative-height image mosaicking. 

## Figures and Tables

**Table 1:** ASTIL echo-imaging and scanning configuration at different flight heights.

Parameter Source	Parameter	2 km	3 km	6 km
Laser unit	Wavelength (nm)	532		
	Pulse repetition frequency (Hz)	2000		
	Nominal fan-beam spatial angle	$\pm 1.5^{\circ }$		
Streak-tube imaging unit	Image resolution	512×1024		
	Temporal resolution	1.29ns/pixel		
	Spatial angular resolution	0.15mrad/pixel		
	Effective echo rows	Approximately 400 rows		
	Instantaneous field of view	$\pm 1.7^{\circ }$		
	Imaging SNR (dB)	25	10	6
Scanning mirror unit	Scan angle range	$\pm 20^{\circ }$	$\pm 26^{\circ }$	$\pm 24^{\circ }$
	Scanning rate (°/s)	20	26	24
	Frame-to-frame scanning step (mrad)	0.349	0.252	0.150

**Table 2:** Geometric characteristics and point spacing of ASTIL scanning swaths at different flight heights.

Flight Height (km)	Swath Length (m)	Swath Width (m)	Point Spacing (m)		
			Flight Direction	Scan Direction, $\phi =0^{\circ }$	Scan Direction, $\left|\phi \right|=\phi_{\max}$
2	1456	104	0.30	0.70	0.79
3	2926	157	0.45	0.76	0.94
6	5343	314	0.90	0.90	1.08

**Table 3:** Image-level acquisition efficiency and output-to-raw data volume ratio of ASTIL relative-height products within a 0.5 s acquisition window.

Parameter	2 km	3 km	6 km
**Common acquisition and image settings**			
Input raw sequence size	1000×512×1024 pixels		
Effective samples for area estimation	1000×350		
Height sampling interval (m)	0.1		
**Altitude-dependent image products and indicators**			
Spatial sampling interval, $\Delta_{s}$ (m)	0.30	0.45	0.90
Estimated image area, $A_{\mathrm{img}}$ (m^2^)	31,500	70,875	283,500
Image acquisition efficiency, $E_{\mathrm{img}}$ (m^2^/s)	63,000	141,750	567,000
Output cropped relative-height image size	500×2313	473×1764	433×1073
Output-to-raw data volume ratio, $\eta_{\mathrm{prod}}$ (%)	0.221	0.159	0.089

**Table 4:** Flight position and attitude stability during the selected ASTIL acquisition windows.

Flight Height	Altitude Std. (m)	Roll Std. (°)	Pitch Std. (°)	Heading Std. (°)	Along-Track Shift (m)
2 km	0.0335	0.0280	0.0238	0.0049	28.46
3 km	0.0091	0.0343	0.0183	0.0154	29.44
6 km	0.0255	0.0168	0.0052	0.0010	27.00

## Data Availability

The data presented in this study are available from the corresponding author upon reasonable request. The full airborne ASTIL dataset is not publicly available due to ongoing research and institutional restrictions.
